# Electrochemical Energy Storage and Conversion Applications of Vanadium Nitrides: Recent Developments and Future Perspectives

**DOI:** 10.1002/smsc.70363

**Published:** 2026-08-02

**Authors:** Warfa Fatima, Sanjana Madapura Suresh, Subhashree Mohapatra, Sang Mun Jeong, Asish K. Kundu, Chandra Sekhar Rout

**Affiliations:** ^1^ Indian Institute of Information Technology, Design & Manufacturing Jabalpur Madhya Pradesh India; ^2^ Centre for Nano and Material Sciences Jain (Deemed‐to‐be University) Bangalore India; ^3^ Department of Chemical Engineering Chungbuk National University Cheongju Chungbuk Republic of Korea; ^4^ Advanced Energy Research Institute Chungbuk National University Cheongju Chungbuk Republic of Korea

**Keywords:** density functional theory, electrocatalysis reactions, energy conversion, energy storage, ion batteries, vanadium nitride, vanadium nitride hybrids

## Abstract

The growing demand for sustainable energy systems has driven advancements in materials for efficient energy storage and conversion technologies. Among emerging electrode materials, vanadium nitride (VN) has attracted attention due to its high electrical conductivity, excellent chemical stability, tunable electronic structure, and abundant electrochemically active sites, making it suitable for many device applications. Synthesis strategies, composite design, and electrolyte selection play critical roles in determining the electrochemical performance of VN‐based materials. This review provides a comprehensive overview of VN, including theoretical insights, various synthesis routes, and their influence on structural and electrochemical properties. The application of VN in energy storage technologies like supercapacitors, ion‐batteries, and metal‐ion capacitors, is systematically discussed, highlighting its high specific capacity, excellent rate capability, and long‐term cycling stability. Furthermore, VN's electrocatalytic performance for hydrogen evolution, oxygen evolution, oxygen reduction, carbon dioxide reduction, and nitrogen reduction is critically evaluated, emphasizing its low overpotentials, enhanced catalytic activity, and favorable reaction kinetics. Despite these advantages, challenges like surface oxidation, structural degradation, and limitations in large‐scale synthesis remain. Future perspectives on nanostructure engineering, heterostructure integration, and advanced electrolyte optimization are also extensively discussed. These characteristics position VN as a significant contender for next‐generation energy storage and conversion technologies.

## Graphical abstract

This review underscores the growing significance of vanadium nitride as a potential material for cutting‐edge energy storage and conversion technologies, attributed to its excellent conductivity, tunable electronic properties, and abundant active sites. It thoroughly examines synthesis methods, theoretical perspectives, and electrochemical applications in supercapacitors, batteries, and electrocatalysis while also addressing current obstacles and future opportunities for next‐generation sustainable energy systems.

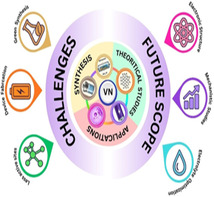

## 1 Introduction

The rapid growth of the global energy sector with increasing environmental concerns associated with fossil fuel consumption, has accelerated the advancement of sustainable and efficient energy storage and energy conversion technologies [1, 2, 3, 4]. In this regard, electrochemical energy systems have emerged as one of the most promising solutions because they enable the direct conversion of electrical energy into chemical fuel and vice versa with improved efficiency, scalability, and environmental compatibility [5]. Furthermore, these systems can be significantly combined with renewable energy sources such as solar and wind power, making them promising candidate for the development of sustainable energy infrastructures [6]. Electrochemical energy storage (EES) systems, including supercapacitors (SCs) and ion batteries [7], as well as electrochemical energy conversion technologies, are primarily dependent on the features of electrode materials and electrolytes. Key characteristics such as electrical conductivity, electrochemical activity, structural stability, ion transport characteristics, catalytic efficiency, and reaction kinetics play crucial roles in determining overall device performance [8, 9, 10, 11, 12]. In energy conversion systems, electrocatalytic reactions such as hydrogen evolution reaction (HER), oxygen evolution reaction (OER), oxygen reduction reaction (ORR), carbon dioxide reduction reaction (CO_2_RR), and nitrogen reduction reaction (NRR) are important because they are directly involved in energy production, storage, and environmental remediation [13]. However, despite their great potential, these electrochemical reactions are frequently restricted by slow reaction kinetics, high overpotentials, poor selectivity, and limited long‐term durability, highlighting the urgent need for advanced electrode and electrocatalytic materials.

Among the wide range of materials investigated, transition metal nitrides (TMNs) have gained remarkable attention due to their unique physicochemical properties, including high conductivity, excellent corrosion resistance, tunable electronic structures, and superior electrochemical activity. Additionally, TMN‐based materials shows high electrical conductivity, large specific capacitance (*C*_sp_), excellent mechanical stability, fast charge–discharge capability, and long cycle life often exceeding 10 000 cycles, making them highly suitable for advanced electrochemical applications [14, 15]. Compared with conventional transition metal oxides, TMNs possess significantly higher electrical conductivity, typically ranging from 4000–55 500 S cm^−1^, which facilitates faster electron transport and improved electrochemical reaction kinetics [16, 17]. In addition, TMNs provide a favorable balance between gravimetric and volumetric capacitance due to their relatively high density and large electrochemically active surface area. Their strong metal−nitrogen bonding also contributes to improved mechanical strength, corrosion resistance, and chemical stability during prolonged electrochemical operation. In view of this, vanadium nitride (VN) has emerged as one of the most potential TMN electrode materials for both EES and energy conversion application because of its high electrical conductivity, favorable electronic structure, and excellent chemical stability. VN exhibits a Pt‐like electronic structure that promotes efficient adsorption and activation of reaction intermediates, making it highly effective for electrocatalytic reactions such as HER, OER, ORR, CO_2_RR, and NRR [18, 19, 20, 21]. In addition to its catalytic activity, VN also demonstrates remarkable pseudocapacitive behavior and rapid electron transport capability, enabling efficient electrochemical charge storage and high‐rate performance. For example, VN nanomaterials have been reported to deliver ultra‐high (*C*_sp_) of up to 1340 F g^−1^ in aqueous electrolytes with outstanding cycling stability and nearly 90% capacitance retention after 1000 cycles [22, 23]. To further enhance the electrochemical performance of VN, extensive efforts have been focused on nanostructure engineering, heterostructure design, and composite formation. VN has been synthesized in various morphologies, including porous nanoparticles, nanosheets (NSs), nanowires (NWs), thin films, and core–shell structures, which provide large electrochemically active surface areas and reduced ion diffusion pathways, thereby improving electrochemical kinetics and active‐site utilization. VN‐based composites combined with conductive carbon materials, polymers, and other transition metal compounds have been extensively studied to enhance electrical conductivity, structural stability, catalytic activity, and overall electrochemical performance. For instance, Feng et al. demonstrated a V–Ni‐based nitride heterojunction (VN/Ni_3_N–Ni/CC) grown on carbon cloth (CC), which achieved a high areal capacitance of 845.63 mF cm^−2^ at 0.5 mA cm^−2^ and retained 86.1% capacitance after 1500 cycles [24]. In addition to conventional SCs, VN‐based materials have also gained considerable attention for microsupercapacitor (MSCs) applications due to their high electrical conductivity and fast redox kinetics driven by the rapid advancement of portable and miniaturized electronic devices. For example, Shen et al. developed a 3D‐printed asymmetric MSC (AMSCs) employing V_2_O_5_ as the cathode and graphene–vanadium nitride quantum dots (G‐VNQDs) as the anode. The device exhibited enhanced energy density (ED) owing to its widened operating voltage window and optimized electrode mass balance. Moreover, the interconnected porous architecture of the 3D‐printed microelectrodes facilitated rapid ion transport and electrolyte diffusion, resulting in enhanced electrochemical performance. The study further suggested that increasing the number of vertically stacked printed layers could further enhance both the energy and power densities of the device [25]. Besides this, VN has been recognized as a potential electrode material for various battery and metal‐ion capacitor (MIC) systems, including lithium‐ion batteries (LIBs), sodium‐ion batteries (NIBs), zinc‐ion batteries (ZIBs), and lithium–sulfur batteries (LSBs), lithium‐ion capacitors (LICs), sodium‐ion capacitors (NICs), and zinc‐ion capacitors (ZICs). Zhang et al. synthesized VN embedded nitrogen‐doped carbon (NC) nanofiber composites with a 3D hierarchical structure, which delivered excellent cycling stability and high‐rate capability for aqueous ZIBs [26]. Similarly, Lu and coworkers designed a Co–VN/NC multifunctional catalyst that enhanced lithium polysulfide adsorption and conversion kinetics, thereby significantly enhancing the electrochemical performance of LSBs [27]. In another study, layered VN derived from V_2_C MXene was employed in NICs, where the hybrid device achieved a high ED of 78.43 Wh kg^−1^ at a power density (PD) of 260 W kg^−1^, showing the strong potential of VN‐based materials for advanced energy storage applications [28].

Beyond energy storage applications, VN‐based materials also exhibit excellent catalytic activity toward several important electrochemical energy conversion reactions. Through enhanced charge transfer and improved active‐site availability, VN significantly improves HER and OER performance in water‐splitting systems [29]. Shen et al. reported that a Ni/VN catalyst exhibited an exceptionally low HER overpotential of 43 mV in 1 M KOH, showing excellent electrocatalytic activity [30]. Similarly, Deepak et al. showed that Cu_2_O/CuO–VN@200W exhibited excellent OER activity with a low overpotential of 190 mV and a Tafel slope of 135 mV dec^−1^ in 1 M KOH [31]. In ORR applications, Huang et al. demonstrated that VN/C catalysts delivered excellent activity in 0.1 M KOH with an onset potential of 0.87 V and a half‐wave potential of 0.73 V [32]. Moreover, VN facilitates the activation of inert molecules such as CO_2_ and N_2_ during CO_2_RR and NRR processes, enabling their conversion into value‐added products while suppressing competing side reactions. These findings demonstrate the outstanding potential of VN‐based materials as efficient and cost‐effective choices over noble‐metal‐based electrocatalysts.

**FIGURE 1:**
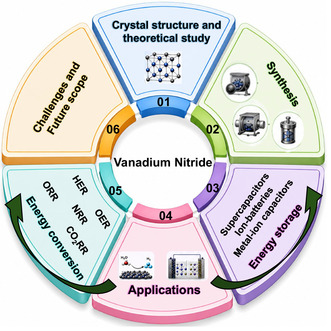
Schematic illustration of the key features of vanadium nitride (VN) and its hybrids discussed in this review work for energy storage and energy conversion applications.

Several review articles have summarized vanadium‐based materials and TMNs for specific electrochemical applications, including aqueous SCs, OER, and TMN‐based energy storage systems. However, these reviews mainly focus on individual applications or broader classes of TMN, without providing a comprehensive discussion of VN from synthesis to multifunctional electrochemical applications. Furthermore, systematic correlations between synthesis strategies, structural engineering, and electrochemical mechanisms remain limited [1, 2, 3, 4, 5, 6, 7, 8, 9]. Unlike previous reviews, the present work provides a comprehensive and integrated overview of VN‐based materials by systematically correlating synthesis strategies, nanostructure engineering, heterostructure design, composite fabrication, and theoretical studies with the EES and energy conversion performance, as illustrated in Figure 1. This review comprehensively summarizes advances in VN‐based materials for SCs, LIBs, NIBs, ZIBs, LSBs, MICs, and electrocatalytic systems, including HER, OER, ORR, CO_2_RR, and NRR. Additionally, the key challenges associated with VN materials, including structural instability, surface oxidation, limited cycling durability, and long‐term catalytic stability, are critically discussed along with several strategies and future perspectives for the next‐generation VN‐based electrochemical materials.

## 2 Crystal Structure and Physical Properties

TMNs are metallic compounds where nitrogen atoms occupy the interstitial sites of the parent metal, having properties akin to ionic and covalently bonded materials. These materials are fascinating for their distinctive electronic structure, exceptional mechanical strength, improved chemical stability, and intriguing electrocatalytic properties. Therefore, TMNs have garnered significant research interest for various applications, particularly in energy storage and conversion [33]. Due to the limited availability of the d‐band and the increased density of states (DOS) near the Fermi level, these TMNs could mimic the behavior of noble metals in electrocatalysis, enhancing the adsorption of reactants and ions on their surfaces. This makes them appealing options for EES and conversion [34, 35]. The notable energy storage capabilities of TMNs could be attributed to the small atomic radius of nitrogen, which leads to a densely packed arrangement of metal atoms within the crystal lattice. This results in better electronic conductivity and stability compared to other transition metal compounds. The covalent bonding between the transition metals and nitrogen atoms increases the lattice cell volume akin to noble metals. Furthermore, nitrogen atoms improve the corrosion resistance of TMNs, making them ideal for use as electrodes in various electrolyte systems [36]. Additionally, when metal nitrides participate in electrochemical or catalytic processes, two significant concepts can be considered. The first concept is the Ligand effect, which suggests that metal nitrides possess an electronic structure distinct from that of the original metal. In these nitrides, the nonmetal gains charge from the metal, leading to hybridization between the metal's d‐states and the nonmetal's sp‐states, as well as an expansion in the metal–metal lattice spacing. This change in electronic structure boosts the chemical reactivity of the original metal, enabling reactants and products to be adsorbed with a strength comparable to that of noble metals, thereby enhancing reaction selectivity. The second concept is the Ensemble effect, where surface nitrogen can significantly reduce the number of available sites on the metal surface. By adjusting the metal‐to‐nitrogen ratio, this effect can be modified. In metal—nitrogen bonding, the d‐band structure of the original metal alters or contracts, which essentially modifies the catalytic site's activity and allows TMNs to exhibit electrocatalytic activity similar to noble metals [37, 38, 39]. Li et al. reported a Co/VN nano‐heterojunction supported on NC, where the combined effects of ligand and ensemble interactions are crucial for its outstanding bifunctional electrocatalytic activity in HER and OER. The ligand effect is due to the directional transfer of charge from electron‐rich Co through VN to the electronegative nitrogen and carbon sites within the carbon matrix. VN acts as an electronic bridge, enhancing the local electronic structure and activating catalytic sites. Simultaneously, the ensemble effect is evident in the creation of a distinct Co/VN heterointerface, which prevents particle aggregation, aids in the uniform distribution of nanoparticles within a 3D porous NS structure and supports efficient mass and charge transport. In the process of OER, the ongoing transformation of VN further reveals and activates cobalt oxyhydroxide species, thereby increasing the number of active sites at the heterojunction. These combined electronic and structural interactions grant the catalyst exceptional activity, with overpotentials of just 116 mV for HER and 311 mV for OER in alkaline conditions [40]. Huang et al. investigated the impact of both electronic and structural adjustments on the ORR performance of vanadium‐based catalysts (VN, V (C, N), VC) by altering nitrogen ratios. The catalyst's electronic structure is greatly affected by the nitrogen‐to‐carbon anion ratio, which determines the electron density at active sites. Incorporating nitrogen enhances electron density at these sites, encourages charge delocalization, narrows the bandgap, and supports a side‐on adsorption configuration for O_2_, thus aiding electron transfer and weakening the O—O bond. At the same time, the reduced radius of nitrogen causes the lattice to contract, affecting the preferential exposure of catalytically active facets. These electronic and structural changes collectively enhance the ORR onset potential and exchange current density as the nitrogen content rises, positioning VN as the most active catalyst among the vanadium‐based options examined [41].

**FIGURE 2:**
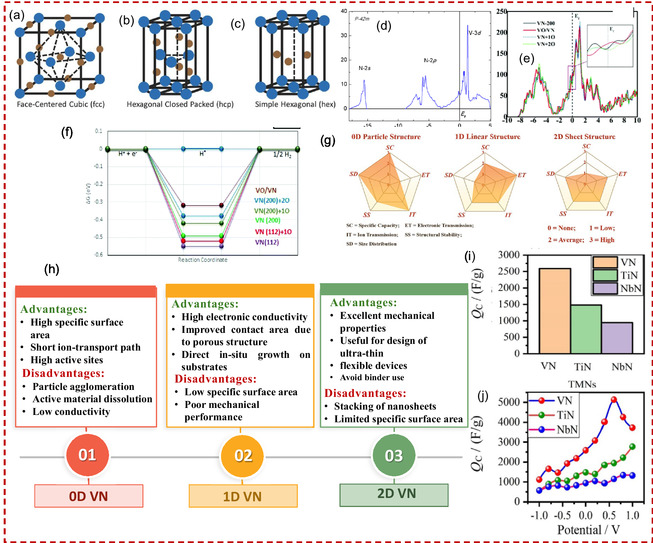
(a–c) Common crystal forms of VN. Reproduced with permission from Ref. [43]. Copyright 2016, Wiley Advanced. (d) Density of states (DOS) as a function of energy for VN. Reproduced with permission from Ref. [44]. Copyright 2016, Elsevier. (e) Comparison between the total DOSs near the Fermi level and (f) comparison between the Gibbs Free energies related to the best catalytic performance of surfaces. Reproduced with permission from Ref. [17]. Copyright 2016, RSC. (g) Distribution of specific capacity, electronic conductivity, ion transport capacity, structural stability, and size distribution among three types of nano VN. Reproduced with permission from Ref. [36]. Copyright 2024, Elsevier. (h) Merits and demerit features of different dimensionality of VN; (i) quantum capacitance (*C*_Q_) calculated for VN, TiN, and NbN at Fermi level done at room temperature; and (j) variation of the *C*_Q_ as a function of operating voltage (bias potential) for VN, TiN, and NbN. Reproduced with permission from Ref. [45]. Copyright 2025, Springer Nature.

Within the TMNs family, VN has gained attention due to its extensive use in alloy components, coatings, battery materials, SC materials, and catalytic carriers. This is attributed to its high melting point, exceptional wear resistance, stability at high temperatures, and excellent electrical conductivity [42]. The vanadium–nitrogen system is characterized by three primary phases: hexagonal close‐packed β‐V_2_N (hcp), face‐centered cubic δ‐VN_1−*x*_ (fcc), and hexagonal δ′‐VN_0.8_ (hex), as illustrated in Figure 2a–c. Among these, the nonstoichiometric mononitride δ‐VN_1−*x*_ (fcc) is noted for its superior stability. The stoichiometry of δ‐VN_1−*x*_ ranges from VN_0.72_ to VN_1_, and this variation significantly influences its physical properties. In view of this, Huber et al. synthesized VN by ammonolysis of ammonium vanadate followed by sintering in NH_3_/N_2_ atmosphere to study its structural, thermal, transport, and magnetic properties [40]. The DOS plot for VN with energy dependence is shown in Figure 2d. The overlap between V‐3d band and N‐2p band occurs at 6 eV below Fermi level (*E*_F_). The cubic structure (rock‐salt type, space group Fm‐3m) of VN is stable at room temperature, but at low temperature (below 205 K) it is stabilized in lower symmetric tetragonal structure (space group P‐42m), with some common fundamental characteristics of the electronic structure. The Fermi level is locked to a peak maximum for cubic (Fm‐3m), however it is shifted away from the maximum by +0.15 eV for tetragonal structure (P‐42m), according to a thorough view of DOS. This might indicate an inherent electrical instability for cubic structure.

Additionally, the tetragonal structure proved to be more stable by about 2 kJ/mol, which is in line with its ground state characteristics. A key property emphasized is its metallic nature, evidenced by low electrical resistivity and a pronounced electronic DOS at the *E*_F_, which underpins its excellent electron transport characteristics relevant to electrochemical applications. The study further reveals a strong electron–phonon interaction, reflected in the thermal transport behavior and specific heat response, alongside a Debye temperature of ∼270 K, suggesting a mechanically robust lattice framework. Importantly, VN also exhibits magnetic characteristics and thermal stability close to ideal stoichiometry, reinforcing its multifunctional character as a material possessing high conductivity, structural tunability, thermal resilience, and electronically active surface states. Those intriguing features hold potential applications for advanced energy storage, catalysis, and electronic device applications [44]. In VN crystal structure, vanadium atoms are in octahedral coordination with nitrogen, resulting in strong metal—nitrogen bonding that imparts both mechanical robustness and high electronic conductivity. The metallic character of VN arises from strong hybridization between V 3d and N 2p orbitals, which create a high DOS at the *E*_F_ and enable admirable electrical conductivity [18]. Ammari et al. investigated the electronic structure of VN, where the analysis of the calculated band structure, DOS, and Fermi surface confirms that VN retains a metallic character, and its phonon spectrum shows no imaginary frequencies across the Brillouin zone, confirming dynamical stability [46]. The first‐principles studies of the broader vanadium–nitrogen phase space similarly found that the low‐energy VN and related V_2_N phases are characterized by broad V 3d—N 2p and V 3d—V 3d bonding bands, consistent with the strong metal‐derived states that dominate near the Fermi level. An additional feature of the calculated electronic structure concerns the effect of nitrogen nonstoichiometry. As nitrogen vacancies are introduced into the VN lattice, vacancy‐induced states appear within the V 3d band, and a local minimum begins to develop in the DOS near the Fermi level. This is accompanied by a systematic decrease in *E*_f_ with increasing vacancy concentration from stoichiometric VN through progressively higher vacancy fractions. Since both electrical conductivity and quantum capacitance scale with *E*_f_, this vacancy‐driven reduction suggests a trade‐off between the thermodynamic stability of nonstoichiometric VN and its intrinsic electronic contribution to charge storage [47]. Hence, the favorable electronic structure and high conductivity of VN support rapid electron transfer during charge–discharge processes, reducing resistive losses and enabling high capacitive performance [43, 48]. The surface chemistry of VN also plays a crucial role in influencing its electrochemical performance and catalytic activity. Unlike bulk VN, which maintains a pure nitride composition, the surface of VN readily interacts with oxygen‐containing species, forming oxy‐nitride or oxide layers. This surface oxidation can occur during synthesis, storage, or electrochemical operation, and its effects on performance are complex and context dependent [43, 48]. Controlled surface oxidation has been shown to enhance electrochemical performance through several mechanisms. First, surface oxide species can participate in reversible redox reactions, contributing additional pseudocapacitance beyond that of the pure nitride [49, 50]. It was exemplified by Choi et al.'s group which noticed impressive results by creating surface nanolayers of transition metal oxides with poor electronic conductivity on the electronically conducting nanocrystalline nitrides [22]. On checking the electrochemical performance, remarkable capacitance does not arise from bulk VN alone, but from a synergistic surface redox mechanism, where an ultrathin electrochemically formed VO_*x*_/oxynitride layer on the metallic VN core undergoes fast and reversible redox transitions in alkaline electrolyte. This conductive core–active shell architecture enables rapid electron transport through the VN core while the surface oxide layer provides multiple reversible oxidation states for charge storage [22]. Second, oxygen‐containing surface groups can improve wettability and electrolyte accessibility, facilitating ion transport to active sites [51]. Third, in electrocatalytic applications, a thin oxide overlayer can modify the electronic structure and adsorption energetics, potentially enhancing catalytic activity for reactions such as hydrogen evolution [18]. To support this, Adimi et al. computed the H adsorption of VN facets and ascertained the effects of oxygen impurities on their catalytic performance by DFT calculations [18]. This provides a way to study the surfaces, model reactions, and quantify the strength of interactions between the catalyst surface and the reaction intermediates. According to DOS graphs, doping oxygen into VN affects the electronic states of nitrogen and vanadium. The total DOS is even slightly raised around the *E*_f_ shown in Figure 2e upon increasing the rate of surface oxidation, indicating improved catalytic activity. The catalytic activity was enhanced when oxygen contaminates the stable VN (112) and (200) facets, especially when the V atoms are in direct contact with oxygen. Therefore, when the uppermost N atoms were replaced with a single vanadium oxide layer on the VN (200) surface, a reduction in the free energy value was observed for VO/VN configuration as seen in Figure 2f. Hence, it was inferred that a single layer of VO acts as a surface oxide activation layer on top of the VN surface, which positively impacts the catalytic performance [18]. These theoretical examinations can guide future experimental investigations and the rational design of high‐performance VN‐based electrocatalysts with controlled oxidation of surfaces. Along with these strategies, nanostructuring of VN based materials, which include 0D VN, 1D VN, and 2D VN, have gained significant attention to enhance the electrochemical/catalytic activity of VN. With change in dimensionality, the physical and chemical properties of VN undergo significant changes as shown in Figure 2g. Each dimensionality of VN possesses certain advantages and demerits, which are summarized in Figure 2h [36].

A high specific capacitance of 1340 F g^−1^, approximately close to theoretical value, was shown by 0D VN owing to its high specific surface area possessing abundant active sites [21]. In contrast, 1D VN has exceptional electron transmission capabilities due to the quantum tunneling phenomenon, which enables some electrons to pass through potential energy barriers at the interface and create a direct electron transmission channel between particles, in comparison to 0D VN. This characteristic increased their electrical conductivity, making them valuable for applications involving energy conversion and storage [52, 53]. In contrast to other nanoforms of VN, the flat planar structure of 2D VN showed significant merits toward the design of flexible device. They showed exceptional stability when they are integrated into flexible SCs as demonstrated by Guo et al.'s group. They created a flexible fiber SC that demonstrated 91% capacitance retention even after 3000 bends by loading VN NSs onto carbon fibers and assembling them with zinc–nickel–cobalt ternary oxide NW arrays [54].

Owing to wide voltage window (∼1.2 V), strong electronic conductivity (∼1.6 × 106 S m^−1^), and high theoretical capacitance (1350 F g^−1^), VN has emerged as a promising contender for use as an electrode material. However, the electrochemical mechanism of VN is based on a rapid surface redox reaction, which limits its energy storage capacity due to the limited surface area. Moreover, the vanadium oxide layer formed on the surface of VN during charging and discharging can dissolve and corrode, further reducing its performance. These two factors are the main reasons why its experimental performance lags behind the theoretical values [36]. The charge storage mechanism of VN has been explained through several phases. Till now the most accepted mechanism considered for VN involves the contribution from both electric double layer and pseudocapacitance reactions which could be expressed as

(I) $${\mathrm{VN}}_{x}O_{y}+{\mathrm{OH}}^{‐}⇌{\mathrm{VN}}_{x}O_{y}||{\mathrm{OH}}^{‐}+{\mathrm{VN}}_{x}O_{y}‐\mathrm{OH}$$

To gain more insights, Liu et al. used in situ Raman and ex situ XRD techniques to examine the charge storage mechanism in the prepared VN materials. It was observed that the valence state of V atom increases during charging, while at discharge time, the cations insert into the crystal structure and the occurrence of redox reactions between hydroxide and V atoms takes place. This shows the combined effect of redox pseudocapacitance and intercalation pseudocapacitance toward charge storage mechanism in VN, which was well supported by theoretical density functional theory (DFT) simulation study [55]. The promising supercapacitive performance of VN was further established from DFT calculations by Rathor et al.'s group. A major finding is the remarkably high quantum capacitance (*C*_Q_) of VN, which reaches 2588 F g^−1^ at the Fermi level and increases to 5135 F g^−1^ at +0.6 V, significantly outperforming TiN and NbN, thereby underscoring its superior charge storage capability from an electronic perspective as shown in Figure 2i. It could be attributed to the high total DOS in the vicinity of the Fermi level for VN compared to other nitrides. For all the nitrides, Figure 2j shows the decreasing trend of *C*_Q_ values for negative biasing, while it increases with increasing the positive bias voltage. Also, there were no significant changes in *C*_Q_ values for these TMNs as temperature varied from −40 to 70 °C, which suggests that *C*_Q_ is thermally stable. In addition, the mechanical analysis reveals that VN possesses good ductility and structural stability, which are crucial for long‐term cycling durability in SC applications [55].

Although the exceptional features of VN endow it to show promising catalytic activity, their relatively low d‐electron occupancy (d^2^ configuration) in V^3+^ ions fundamentally weakens the adsorption capacity of oxygen intermediates (*OOH/*O species), as evidenced in V‐based single‐atom catalyst systems. This inherent electronic configuration leads to compromised ORR/OER bifunctional activity, thus limiting the implementation in metal–air battery systems. To overcome it, both experimental and theoretical investigations indicate that it is possible to modify the chemical environment of the V active site by doping with a different transition metal, thereby enhancing the catalytic activity and stability of the catalyst. In this regard, Li et al. used Co as a dopant in VN due to its unique filling states and strong electron‐supplying capability. The Co doping technique efficiently controls the adsorption/desorption equilibrium of ORR intermediates through d‐electron transfer to V atoms while dramatically reducing the activation energy barrier of the rate‐determining phase, according to DFT analysis. The modified V_0.95_Co_0.05_N/NC/C catalyst obtained a low bifunctional index (Δ*E* = 0.78 V) electrochemically with a half‐wave potential for the ORR of 0.83 V and an OER overpotential of 380 mV at 10 mA cm^−2^. It achieved a high specific capacity (708.98 mAh g^−1^) in Zn–air batteries and maintained 88% of the starting voltage after 280 h of cycling. These results demonstrated the potential of a doping method to modify the d‐band centers in TMNs for energy conversion devices [56]. Additionally, TMNs have shown significant potential for usage in LIBs, but their catalytic activity and lithophilic properties are subject to the contraction of the d‐orbitals in the metal center. To modulate the electronic structure of TMNs, Liu et al. adopted heterojunction engineering to construct a p–n heterojunction catalyst by incorporating p‐type iron nitride and n‐type VN (p‐Fe_2_N/n‐VN) heterostructures embedded in vesicle‐shaped N‐doped nanofibers. It was found that the high‐spin Fe atom's d‐band center absorbs more electrons from the V atom to achieve more π* and moderate σ* bond electron filling and orbital occupation. This allows for more efficient d–p orbital hybridization to enhance reaction kinetics and moderate adsorption intensity for polysulfides. Such regulated electronic structure inhibits the shuttle effect; thus, improving the transformation kinetics and inhibiting the growth of Li dendrites [57].

**FIGURE 3:**
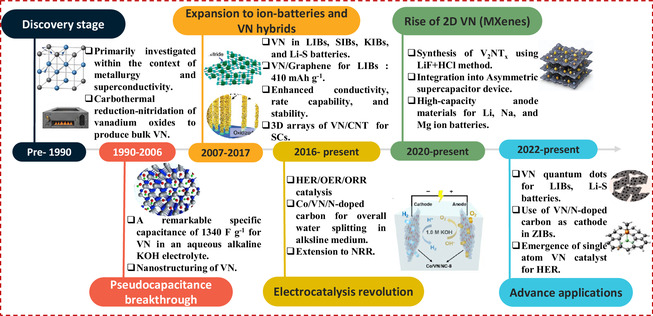
Timeline on evolution of VN for energy storage, ion‐batteries, and catalytic applications.

Figure 3 depicts the gradual transformation of VN from a refractory ceramic into a cutting‐edge multifunctional platform for electrochemical energy storage and conversion. Initially, VN was recognized for its metallic conductivity and thermal stability, which was followed by a breakthrough in pseudocapacitance that highlighted its outstanding charge‐storage potential for SCs. Later advancements concentrated on VN hybrids and nanostructured composites for lithium‐, sodium‐, and potassium‐ion batteries, which improved conductivity, ion transport, and cycling durability. The development of 2D VN MXenes further broadened its use in high‐rate energy storage devices. Recently, VN‐based materials have shown impressive electrocatalytic activity for HER, OER, ORR, and NRR through defect engineering and heterointerface design. Current research is focused on advanced applications such as single‐atom VN catalysts, VN quantum dots, and integrated self‐powered energy systems, establishing VN as a potential material for future sustainable energy and conversion technologies.

## 3 Synthesis of VN

**FIGURE 4:**
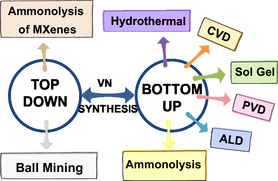
Overview of top‐down and bottom‐up synthesis strategies for VN.

The synthesis of VN has been achieved using both top‐down and bottom‐up approaches, each influencing the structural and electrochemical properties of the final material. Top‐down methods, including mechanical activation and chemical etching techniques, are effective for producing VN thin films and layered nitride structures, but offer limited control over nanoscale morphology and porosity, which are critical for electrochemical applications. To overcome these limitations, bottom‐up synthesis strategies, which construct materials from atomic or molecular precursors, have been widely adopted due to their ability to precisely control composition, structure, and morphology. Figure 4 shows the schematic illustration of top‐down and bottom‐up approaches for the synthesis of VN. The following section focuses on bottom‐up approaches for the synthesis of VN and VN‐based nanostructures.

### 3.1 Bottom‐Up

The bottom‐up approach in materials synthesis refers to the synthesis of materials starting from the atomic or molecular building blocks, which subsequently assemble into nanostructures through chemical reaction or self‐organization processes. This strategy enables precise control over composition, crystal structure, morphology, defect density, and surface chemistry [58]. Bottom‐up techniques include ammonolysis, sol–gel, hydrothermal/solvothermal, chemical vapor deposition (CVD), atomic layer deposition (ALD), and magnetron sputtering methods that have been used for VN synthesis.

#### 3.1.1 Ammonolysis/Thermal Nitridation of Oxides

Ammonolysis refers to a high‐temperature chemical process in which a precursor reacts with ammonia at elevated temperatures. This method is widely used for the synthesis of nitrides, where the oxide, halide, and sulfide precursors are exposed to a flowing ammonia atmosphere under controlled heating. During this process, ammonia acts as a reducing and nitriding agent: it reduces the precursor (e.g., oxide to water) while simultaneously supplying nitrogen, leading to the formation of metal nitrides [59]. During the process, the precursor's external morphology is the same, but its internal structure becomes porous, which is preferred for energy storage applications [35]. Many studies have reported the synthesis of TMNs via ammonolysis. For example, Gao et al. synthesized Mo_2_N and V_2_N through the ammonolysis of Mo_2_CT_*x*_ and V_2_CT_*x*_ MXenes at 600 °C, marking the first nitride MXene formation [60]. Similarly, mesoporous Mo_3_N_2_ NWs have been prepared via nitridation of MoO_3_ NWs [61], while 2D MoN NSs were obtained by nitriding h‐MoO_3_ precursors [62]. In addition, various nanostructures of VN, TiN, and Nb_4_N_5_ with controlled morphologies have been successfully synthesized through the nitridation of corresponding transition metal oxides [60, 61, 62]. For example, Bi et al. synthesized VN mesocrystal nanoshells via nitriding a layered Na_2_V_6_O_16_, and Gao et al. prepared mesoporous VN NWs by thermally nitriding V_2_O_5_ NWs. Notably, the resulting nitride phase exhibits higher density and reduced volume relative to the precursor oxide [35] which affects the structural integrity and electrochemical performance of the material. Ammonolysis is one of the most widely adopted routes for VN synthesis because of its simplicity, ability to preserve precursor morphology, and capability to produce nanocrystalline structures that are highly beneficial for electrochemical applications. For instance, urea‐assisted nitridation has been reported to produce mesoporous VN with a surface area as high as 57 m^2^ g^−1^ [63]. However, this method is limited by the need to handle NH_3_ gas at elevated temperatures, the possible formation of residual amide/imide species, and incomplete nitridation at lower temperatures. From a manufacturing perspective, prolonged heat treatment and stringent ammonia‐handling requirements present challenges for large‐scale production. Nevertheless, furnace‐based ammonolysis remains a potentially scalable route for bulk powder synthesis [64]. Although the precursor materials are relatively inexpensive, energy consumption and controlled ammonia flow increase the overall operating cost.

#### 3.1.2 Sol–Gel

The sol–gel process is a solution‐based chemical synthesis technique in which metal salts or metal alkoxides precursors are dissolved in a suitable solvent. In the hydrolysis stage, the metal alkoxides interact with water, producing metal hydroxyl species as represented by the following reaction

(II) $$M–\mathrm{OR}+H_{2}O\to M–\mathrm{OH}+\mathrm{ROH}$$

This is followed by a condensation process in which hydroxyl groups combine to form metal—oxide—metal bonds (M—O—M) with the release of water

(III) $$M–\mathrm{OH}+M–\mathrm{OH}\to M–O–M+H_{2}O$$

As a result, the sol stage consists of numerous hydrolyzed metal species and partially condensed (M—O—M) clusters that are dispersed as colloidal particles [65]. Many transition metal oxides have been effectively prepared through the sol–gel synthesis method, owing to the composition control and ability to produce homogeneous materials at relatively low temperatures. For example, Cui et al. prepared TiVN/carbon composites with varying V/Ti ratios via sol–gel technique. Several reports have further demonstrated the versatility of this method. These include the synthesis of needlelike VN nanostructures [66], VN obtained via a sol–gel reaction of vanadium alkoxides with urea [66], and porous VN nanoribbon/graphene composites [67], which exhibit promising performance as cathode materials for LSBs. In addition, Kiruthiga et al. reported the synthesis of V_2_O_5_@rGO nanorods by integrating sol–gel–derived V_2_O_5_ with rGO prepared using a modified Hummers’ method [68]. These studies collectively highlight the significance of sol–gel method in fabricating nitride‐based nanostructures with tunable morphologies and improved electrochemical performance [69]. The sol–gel method offers excellent compositional homogeneity, and its solution‐based processing is relatively inexpensive and can be carried out using conventional laboratory equipment [66]. However, sol–gel synthesis generally serves as a precursor preparation route rather than a direct nitridation method, requiring an additional heat treatment and ammonolysis step to obtain VN. Owing to its simplicity, low precursor cost, and compatibility with large‐batch processing, the method is considered highly promising for scalable production [63].

#### 3.1.3 Hydrothermal/Solvothermal Synthesis

The hydrothermal/solvothermal method is a facile wet‐chemical approach widely used for synthesizing nanoparticles with controlled morphology [70]. In this process, a precursor is dissolved in a solvent and subsequently recrystallized into the desired form under controlled temperature and pressure conditions in a sealed system [58]. The main difference between hydrothermal and solvothermal synthesis methods is the type of solvent employed; hydrothermal synthesis uses water as the reaction medium, whereas solvothermal synthesis utilizes nonaqueous or organic solvents [71]. This technique extensively adopted due to its ability to precisely control crystal structure, morphology and phase purity by tuning parameters such as pressure, temperature, and reaction time [65, 70]. Compared to other synthesis methods, it typically operates at moderate temperatures (130–250 °C) and pressure (0.3–4 MPa) [65]. A broad variety of morphologies including NWs, nanorods, NSs, nanospheres, and nanoribbons can be obtained using this approach [58]. Various vanadium‐based materials have been synthesized via hydrothermal/solvothermal methods, including VO_2_ NSs [65, 72], VO_2_(B) hollow spheres [73], V_2_O_3_@C core–shell nanorods [74], porous VN NWs [75], NiCo_2_S_4_@VS_2_ [76], and Co_3_V_2_O_8_–Ni_3_V_2_O_8_ nanocomposites [77]. Several studies have also reported the growth of VN NWs on CC via nitridation of hydrothermally synthesized oxide NWs [35, 75]. In addition, hierarchical carbon‐coated porous VN NSs have been fabricated through a hydrothermal‐assisted route, where V_2_O_5_ NSs were first grown on a conductive carbon framework via hydrothermal treatment, followed by ammonia nitridation and subsequent carbon coating. This strategy results in interconnected porous VN NSs with enhanced ion diffusion and electrical conductivity, leading to enhanced electrochemical performance, such as high (*C*_sp_), strong rate performance, and excellent cycling stability [15]. Overall, this approach has been extensively utilized for fabrication of electrode materials for batteries, SCs, electrocatalysis, and sensing applications, highlighting its versatility in energy storage and conversion technologies. Despite its advantages in controlling morphology, particle size, and crystallinity, hydrothermal synthesis of VN generally requires a subsequent nitridation step using NH_3_ to achieve complete conversion of oxide precursors into VN. Furthermore, autoclave‐based batch processing limits continuous large‐scale production. Consequently, the hydrothermal approach is generally regarded as a moderately scalable technique with moderate production costs [41, 78].

#### 3.1.4 CVD

CVD is a commonly employed industrial technique for synthesizing solid materials through gas phase reactions. In this process, one or more gaseous precursors undergo chemical reaction or thermal decomposition occurring either within the gas phase or at the gas–solid boundary, resulting in the formation of a solid film on a heated substrate [79]. Several variants of CVD have been developed, including atmospheric‐pressure CVD, low‐pressure CVD, ultrahigh‐vacuum CVD, laser‐assisted CVD, metal–organic CVD, and plasma‐enhanced CVD, each operating under specific conditions such as pressure, gas flow rate, and energy source [65]. The quality and uniformity of the deposited film are governed by multiple parameters, including the partial pressure and relative ratio of the reactant gases, deposition temperature, nature of the substrate, total system pressure, gas flow rate, reactor configuration, gas delivery system materials, and the purity of precursor compounds [65].

Under controlled conditions, CVD techniques can produce a diverse range of materials, including oxides, sulfides, nitrides, borides, dichalcogenides, and carbon‐based nanostructures [58, 80]. Among these, metal–organic CVD, which employs organometallic precursors, is particularly important for the synthesis of TMNs and vanadium‐based thin films [65]. For example, Chakrapani et al. used a hot‐filament chemical vapor deposition (HFCVD) method combined with a nitridation process to synthesize 2D TMNs [58, 81]. Similarly, Yu et al. synthesized VN–graphene foams via CVD using Ni foam as the growth substrate and methane as the carbon source, achieving uniform hybrid architectures with high conductivity [82]. In addition, Basu et al. reported the synthesis of a nanoporous VO_2_ structure on carbon paper substrates via a directed vapor transport growth process [83]. The major advantages of CVD include the deposition of high‐quality crystalline VN thin films with excellent control over morphology, crystal orientation, and substrate integration. However, the process is sensitive to precursor purity and delivery conditions and is susceptible to oxygen contamination during growth. Although CVD requires sophisticated equipment and relatively high capital investment, it remains a well‐established and scalable technique for industrial coatings and semiconductor manufacturing [84].

#### 3.1.5 Magnetron Sputtering

Magnetron sputtering is a physical vapor deposition (PVD) method in which a metal target acting as a cathode (e.g., vanadium) is bombarded with energetic ions (Ar^+^ and N^+^/N_2_^+^) generated in an argon/nitrogen plasma, resulting in the ejection of metal atoms and secondary electrons [58]. A magnetic field is imposed parallel to the target surface to confine electrons near the cathode, thereby enhancing plasma density and ionization efficiency. The ejected metal atoms subsequently react with activated nitrogen species to form metal nitride films, which condense on the substrate [85, 86]. This method offers strong film adhesion, precise control over composition and thickness, and favorable microstructural properties. The presence of magnetic field increases the electron path length and collision probability, enabling high plasma density at relatively low operating voltages (typically 500–600 V), which promotes efficient film growth [87]. Magnetron sputtering has been widely applied for the synthesis of TMNs, enabling control over surface morphology, porosity, and electrochemical properties [58]. For example, oblique angle magnetron sputtering has been employed for the preparation of porous CrN thin films with a controlled surface roughness of 32.95 nm [88]. Similarly, Wei et al. synthesized CrN by selectively etching Cu from sputter‐deposited CrCuN thin films using nitric acid, significantly increasing surface roughness after Cu removal [89]. For VN synthesis, argon is commonly used as the sputtering gas, while nitrogen serves as the reactive gas, and the final film composition can be effectively controlled by adjusting the total pressure and Ar/N_2_ gas ratio [90]. For instance, Zhang et al. deposited VN layers onto carbon nanotube arrays deposited onto glassy carbon or inconel substrates using magnetron sputtering, achieving enhanced electrochemical performance [35]. Early studies by Lúcio‐Porto et al. demonstrated that a VN film of 25 nm thickness delivered a high capacitance of 422 F g^−1^ in 1 M KOH, highlighting the importance of thickness control [91]. Subsequent work by Bondarchuk et al. showed that sputtering conditions such as film thickness (100–400 nm), gas composition, and oxygen‐free environments significantly influence electrochemical behavior, and suggested space–charge accumulation as a substitute for conventional charge storage processes [92, 93]. Overall, magnetron sputtering is an efficient method for the synthesis of VN thin‐film electrodes because of its precise controllability, scalability, and capability to generate high‐quality nitride films [58]. Magnetron sputtering is among the most industrially compatible techniques for VN thin‐film fabrication, offering excellent control over film composition, thickness, density, and microstructure [94]. However, the quality of deposited films is highly sensitive to processing parameters such as nitrogen flow rate, chamber pressure, and target condition. Furthermore, increasing nitrogen content may reduce the deposition rate and induce target poisoning effects [95]. Although the equipment cost is relatively high, magnetron sputtering is highly scalable for large‐area coating applications and is considered economically attractive for industrial production [96].

#### 3.1.6 ALD

ALD is a vapor‐phase thin‐film deposition technique that provides accurate thickness regulation and highly uniform coating, making it particularly suitable for fabricating TMN thin films. For VN synthesis, a vanadium‐based precursor is first introduced into the deposition chamber, where it adsorbs selectively onto the substrate surface, forming an ultrathin vanadium‐containing layer. Subsequently, an inert gas such as argon is purged through the chamber to remove excess precursor species and byproducts. A nitrogen source is then introduced, which reacts with the adsorbed vanadium layer to form a thin VN film. The thickness can be accurately controlled by repeating these deposition cycles. This sequential and self‐limiting nature of ALD enables precise control over the thickness and uniformity of VN thin films [35]. For instance, Kozen et al. presented the direct growth of VN thin films using plasma‐enhanced ALD (PEALD), in which a metal–organic vanadium precursor, such as tetrakis (dimethylamido) vanadium, was combined with nitrogen plasma. The plasma provides highly reactive nitrogen species, enabling in VN deposition at relatively low temperatures. The use of nitrogen plasma was shown to improve film density and reduced impurities [97]. Apart from direct ALD for VN synthesis, the technique is often coupled with postdeposition ammonolysis, wherein ALD‐deposited vanadium oxide films are subsequently converted into VN through a nitridation process [35].

ALD provides exceptional control over film thickness, composition, and conformality, making it particularly attractive for microelectronic and nanoscale device fabrication. However, the requirement for highly specialized metal–organic precursors, low deposition rates, and expensive instrumentation limits its large‐scale industrial implementation. Consequently, ALD is primarily employed for high‐value applications where precise film quality is more important than production throughput [84, 98].

### 3.2 Top‐Down Synthesis

The top‐down approach in materials synthesis refers to the conversion of large‐scale materials into lower‐dimensional structures through physical or chemical process. Although widely employed, achieving precise control over structure and morphology remains challenging. In this approach, techniques such as mechanical activation and chemical etching are used to break down strongly bonded bulk phase. For VN, top‐down synthesis has been demonstrated through mechanochemical routes, particularly reactive high‐energy ball milling under a nitrogen atmosphere, where bulk vanadium is directly converted into VN. During milling, repeated high‐energy collision introduces lattice defects, grain refinement, and internal strain. These effects continuously generate fresh and highly reactive vanadium surfaces, facilitate nitrogen diffusion, and nitride formation without the need for external high temperature treatment [99, 100].

Although high‐energy ball milling can produce VN nanoparticles rapidly, even at room temperature, it suffers from several limitations, including contamination from the milling media, the requirement for high‐energy milling or pressurized nitrogen environments, and purity levels that are sometimes lower than those achieved using conventional nitridation methods [99, 101]. Nevertheless, this approach is well suited for large‐scale powder production and is considered more cost‐effective than advanced thin‐film deposition techniques such as ALD and CVD [102].

In addition to mechanochemical methods, VN can also be synthesized via a MXene‐derived top‐down transformation route. In this approach, carbide MXene (V_2_CTx), produced through the selective removal of the Al layer from the V_2_AlC MAX phase, is subjected to ammoniation at elevated temperature (typically 600 °C) in an NH_3_ atmosphere. During this process, the layered V_2_CTx undergoes nitridation, where nitrogen atoms progressively replace carbon, leading to the formation of VN or mixed V_2_N/VN phases while maintaining most of the original 2D layered structure. This structure‐retaining conversion results in high surface area and enhanced electrochemical accessibility, distinguishing it from conventional bottom‐up synthesis routes [60]. Beyond direct nitride formation, V_2_N MXene can also be synthesized via a top‐down approach by selectively etching the A layer from the parent MAX phase, leaving behind loosely stacked M–X layers that can be further delaminated into few‐layer sheets [103, 104]. However, etching in nitride‐based systems is more challenging than in carbides, as conventional HF‐based etchants are less effective due to the higher stability of nitride MAX phases. To address this, alternative acid–salt etching systems such as NaF–HCl and LiF–HCl are employed to selectively etch the Al layers while preserving the metal–nitrogen framework [94, 95]. In a typical process, fluoride salts (e.g., NaF or LiF) are dissolved in hydrochloric acid to form the etchant, followed by introduction of the layered precursor. The reaction is maintained at elevated temperature for extended durations to ensure complete etching. The obtained product is repeatedly rinsed with deionized water until a neutral pH is reached, followed by vacuum drying [105, 106]. To obtain few‐layer V_2_NT_*x*_ MXene, intercalation and delamination steps are carried out using agents such as dimethyl sulfoxide (DMSO) or tetrabutylammonium hydroxide (TBAOH), followed by mild sonication [107]. This process yields thin, loosely stacked V_2_N MXene sheets with enhanced surface accessibility. Subsequent characterization studies have confirmed their metallic conductivity and favorable electronic properties, further supporting their potential in electrochemical applications. MAX‐phase etching is primarily employed for the synthesis of 2D V_2_N MXene rather than bulk VN. Its principal advantage lies in producing delaminated 2D NSs with high surface area and excellent electrical conductivity. However, the process is inherently multistep, requiring MAX‐phase synthesis, selective etching, intercalation, and delamination. These chemically intensive processing steps increase both the complexity and overall production cost, limiting the scalability of this approach [107]. Overall, sol–gel‐assisted nitridation and mechanochemical synthesis appear to be the most cost‐effective and scalable routes for bulk VN powder production, whereas magnetron sputtering is the preferred industrial technique for large‐area VN thin films. CVD and ALD provide superior control over film quality and thickness, making them suitable for high‐performance electronic applications despite their higher cost. In contrast, MAX‐phase etching remains a specialized route for producing 2D V_2_N MXenes rather than a cost‐effective approach for bulk VN synthesis. Table 1 summarizes the existing synthesis strategies and the corresponding morphologies of VN and VN‐based composite materials, highlighting the diversity of fabrication approaches employed to tailor their structural characteristics for electrochemical applications.

**TABLE 1:** Reported synthesis strategies for vanadium nitride (VN) and VN‐based composite materials.

Material	Morphology	Synthesis method	Reference
VN nanoparticles	Nanoparticles	Ammonolysis	[22]
Porous nanocrystalline VN	Porous nanocrystals	Ammonolysis	[48]
3D VN–CNT arrays	3D array	Hydrothermal + ammonolysis	[108]
Spherical porous VN	Porous spheres	Template‐assisted ammonolysis	[109]
TiN/VN composite	Thin film/composite	Magnetron sputtering	[110]
Mesoporous TiN–VN core–shell fibers	Core–shell fibers	Electrospinning + ammonolysis	[49]
VN nanodots in carbon nanosheets	Nanodots/nanosheets	Hydrothermal	[111]
Carbon‐coated porous VN	Porous nanoparticles	Hydrothermal + ammonolysis	[15]
Porous VN thin films on silicon wafer	Thin film	Solution‐based synthesis	[112]
VN/PEDOT core–shell	Core–shell	Wet chemical method	[113]
Fiber‐shaped VN@C device	Fiber	Hydrothermal + ammonolysis	[108]
VN/porous carbon nanoparticles	Nanoparticles	Sol–gel + pyrolysis	[114]
VN	Thin film	CVD	[98]
VN/C	Nanocomposite nanoparticles	Sol–gel	[115]
VN	Bulk crystalline	Solid‐state synthesis	[116]
VN nanoparticles	Nanoparticles	Rapid heating method	[64]
VN nanocrystals	Nanocrystals	Thermal decomposition + ammonolysis	[117]
VN	Particles	Microwave‐assisted + ammonolysis	[42]
VN	Particles	Ammonolysis	[118]
VN complexes	Molecular complexes	Chemical synthesis	[119]
VN/V_2_N 2D nitrides	2D nanosheets	MXene‐derived ammonolysis	[60]
Pea‐shaped VN nanorods	Nanorods	Hydrothermal + ammonolysis	[120]

## 4 VN for Energy Storage Applications

The rapid expansion of renewable energy sources, coupled with increasing global energy consumption, has increased the demand for efficient energy storage systems that deliver ED and PD along with good safety, cost‐effectiveness, long cycling stability, and environmental sustainability [59, 121]. Consequently, significant research efforts have been directed toward advanced EES devices, such as batteries, SCs, and metal ion capacitors. These systems typically consist of three key components: a cathode, an anode, and an electrolyte that facilitates ion transport [122, 123], and they operate via distinct charge storage mechanisms [124, 125, 126]. Batteries mainly store energy through faradaic redox reactions occurring in the electrode materials, resulting in high ED. In contrast, SCs exhibit high PD, fast charge–discharge capabilities, and excellent cycling stability, while metal ion capacitors bridge the gap between these two systems by combining high ED with high power performance. The overall effectiveness of these devices is largely governed by the electrochemical activity, structural stability, electrical conductivity, and ion transport characteristics of the electrode materials [124]. Accordingly, numerous electrode materials have been extensively studied, including carbon‐based materials [16, 123, 127], conducting polymers (CPs) [59], transition metal oxides [16], sulfides/selenides [13, 126, 127], metal carbides [128], and metal nitrides [19, 55]. These materials exhibit desirable properties such as high reversibility, good safety, short ion diffusing paths, excellent electrical conductivity, and superior surface reactivity, which makes them a suitable candidate for EES systems [17]. Among these electrode materials, TMNs including TiN [127, 129], Mo_2_N [128], NbN [130], FeN [131], Ni_3_N [132], Ta_3_N [129], and CrN [133], have attracted considerable attention due to their high metallic conductivity, good chemical and thermal stability, and favorable electronic structures [134]. As a result, they demonstrate high *C*_sp_, excellent rate capability, and stable cycling performance. However, challenges such as brittleness, limited active sites, and durability issues still restrict their practical application [35, 59]. Within this class, VN has emerged as a particularly promising electrode material for advanced EES system [78]. VN exhibits enhanced charge storage capability and rapid charge–discharge behavior thereby making them a potent material for developing efficient energy storage devices [23].

### 4.1 Supercapacitors

**FIGURE 5:**
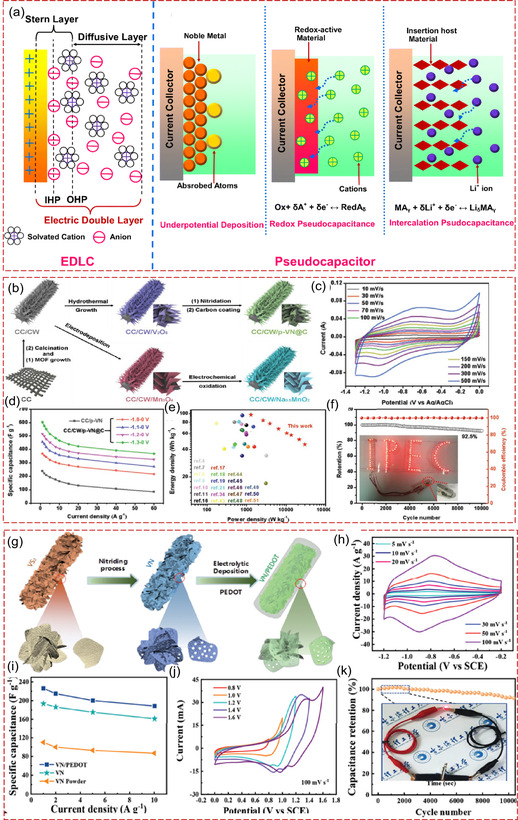
(a) Charge storage mechanisms in supercapacitors. Reproduced with permission from Ref. [140]. Copyright 2023, RSC. (b) Schematic fabrication hierarchical carbon‐supported VN nanosheet/wall electrodes structured CC/CW/p‐VN@C anode and CC/CW/Na_0.__5_MnO_2_ cathode; (c) CV; (d) *C*_sp_ different current densities of the CC/CW/p‐VN@C electrode; (e) Ragone plot; and (f) cycling stability (inset shows device demonstration) of the CC/CW/p‐VN@C||CC/CW/Na_0.__5_MnO_2_ ASC system. Reproduced with permission from Ref. [15]. Copyright 2019, Wiley Advanced. (g) Schematic illustration of the synthesis process for VN/PEDOT core–shell nanoarrays; (h) CV; (i) *C*_sp_ at different current densities of the VN/PEDOT electrodes; (j) CV; and (k) cycling stability of the flexible VN/PEDOT ASC device (inset shows flexible device demonstration). Reproduced with permission from Ref. [113]. Copyright 2020, Wiley Online Library.

SCs are widely investigated EES systems that store charge mainly through two mechanisms: electric double‐layer capacitance (EDLC) and pseudocapacitance [4, 135, 136]. Both processes occur at the electrode–electrolyte interface but differ fundamentally in their charge storage behavior [137]. In EDLCs, energy storage occurs through the electrostatic accumulation of ions at the electrode–electrolyte interface without electron transfer, resulting in non‐faradaic interactions [138, 139]. Upon application of a potential, excess charge accumulates on the electrode surface, attracting oppositely charged ions from the electrolyte and forming an electric double layer consisting of a compact Helmholtz layer or stern layer and a diffuse Gouy–Chapman layer, as shown in Figure 5a [141]. The capacitance is governed by surface area and charge separation distance and can be expressed as

(1) $$C=\frac{\text{ε}A}{d}$$

where *C* represents the double‐layer capacitance, *ε* denotes the permittivity of the dielectric medium separating the charges, *A* corresponds to the effective electrode surface area, and *d* refers to the distance separating charges at the electrode–electrolyte interface (typically in the nanometer range) [142]. Due to this small charge separation distance and large accessible area, SCs are reported to store up to 10^5^ times more energy than conventional capacitors [143]. In contrast, pseudocapacitors store energy via rapid and reversible faradaic redox reactions taking place on or near the electrode surface [141, 144]. Despite involving electron transfer, these system exhibit capacitor‐like electrochemical behavior such as quasirectangular cyclic voltammetry (CV) curves and nearly triangular galvanostatic charge–discharge (GCD) profiles [141, 144]. The active region is typically confined to a shallow depth proportional to (2*Dt*)^1/2^, where *D* represents the diffusion coefficient and *t* denotes the diffusion time [144]. Such pseudocapacitive behavior is typically exhibited by TMNs, CPs, transition‐metal oxides/hydroxides, transition‐metal dichalcogenides (TMDs), and MXenes. In contrast, EDLC behavior is commonly observed in high‐surface‐area materials such as activated carbon, carbon nanotubes, graphene, and other carbon‐based materials. Studies have reported that EDLCs respond more quickly to the changes in applied potential than pseudocapacitors, owing to their rapid rearrangement of charges during EES [140, 144].

Among the various pseudocapacitive materials, TMNs have gained significant attention due to their metallic conductivity and fast redox kinetics. Among them, VN has emerged as a highly promising electrode material for SC applications. Compared with other metal nitrides, VN demonstrates high conductivity (*σ *≈ 1.17 – 1.67 × 10^6^ S m^−1^), relatively low cost, and a wide potential window, making it a good candidate for advanced EES devices with both high energy and power capabilities. Unlike carbon‐based materials that primarily store charge through EDLCs, VN exhibits pseudocapacitive behavior due to the rapid and reversible surface faradaic reactions. The electrochemical behavior of VN electrodes is strongly affected by the type and composition of the electrolyte used, which governs the capacitance, operating potential window, reaction kinetics, and long‐term stability of the device. Several studies have demonstrated that the charge storage mechanism in VN generally involves both EDLC and surface faradaic redox reactions. Quantitative analysis by Djre et al. confirmed that pseudocapacitance dominates the overall charge storage, contributing approximately 85% in basic electrolyte and 87% in acidic electrolyte [145].

In most VN electrodes exposed to ambient conditions, a thin layer of surface oxides is formed, leading to formation of vanadium oxynitride species (VN_*x*_O_*y*_). These oxynitride species actively participate in reversible redox reactions during electrochemical cycling. Consequently, the charge storage mechanism in VN is generally described as a combined effect of EDLC and surface faradaic reactions occurring on partially oxidized oxynitride surfaces. In alkaline media, the widely accepted reaction mechanism is expressed as

(IV) $${\mathrm{VN}}_{x}O_{\gamma }\;+\;{\mathrm{OH}}^{‐}\;↔\;{\mathrm{VN}}_{x}O_{\gamma }//\mathrm{OH}\;+\;{\mathrm{VN}}_{x}O_{\gamma }–\mathrm{OH}$$

which represents the reversible interaction between hydroxyl ions and surface oxynitride species [146]. This process enables rapid and reversible redox reactions occurring at the electrode surface contributing significantly to the pseudocapacitive behavior of VN. In addition to this oxynitride‐mediated mechanism, valence changes in vanadium ions have also been proposed to contribute to charge storage. A representative redox reaction describing the valence transition is expressed as

(V) $$V^{3+}N+2{\mathrm{OH}}^{‐}+e^{‐}↔V^{2+}{N(\mathrm{OH})}_{2}$$

which further confirms the involvement of reversible surface redox reactions during electrochemical cycling [147]. Among different electrolytes, alkaline electrolytes such as KOH are widely used for VN‐based SCs because they provide favorable reaction kinetics and a wider operating potential window. In concentrated KOH solutions, the transformation overpotential between different vanadium oxidation states is significantly reduced, enabling faster redox reactions and improved electrochemical performance [59]. In contrast, under neutral or acidic electrolytes, the charge storage mechanism is different and can involve ion intercalation processes described by

(VI) $$V_{\left\{x+2y/3\right\}}N_{x}O_{y}+zM^{+}+ze^{‐}↔V_{\left\{x+2y/3\right\}}N_{x}O_{y}M_{z}(M^{+}={\mathrm{Li}}^{+},H^{+})$$

where electrolyte cations are reversibly inserted into the VN structure. However, the electrochemical stability in such electrolyte is limited to 0.2–0.6 V, which is significantly narrower than that achievable in alkaline systems [148]. Apart from aqueous electrolytes, several studies have also explored gel and nonaqueous electrolytes to improve the cycling stability of VN electrodes. For instance, Lu et al. reported that VN NWs in a LiCl/PVA gel electrolyte delivered a *C*_sp_ of 298.5 F g^−1^ at 10 mV s^−1^, with 95.3% capacitance retention after 10 000 cycles, which was significantly higher than the 14.1% retention observed in 5 M LiCl aqueous electrolyte [59]. This enhancement was attributed to the protective effect of the gel electrolyte, which minimizes structural degradation during repeated cycling. Furthermore, Wang et al. investigated the use of LiPF_6_ electrolyte, commonly employed in LIBs, and proposed a lithium insertion reaction of the form

(VII) $$\mathrm{VN}+x{\mathrm{Li}}^{+}+xe^{‐}↔{\mathrm{Li}}_{x}\mathrm{VN}$$

suggesting that VN can also exhibit lithium storage behavior under appropriate electrochemical conditions [149]. These findings highlight that the electrolyte selection plays a vital role in controlling the charge storage mechanism and electrochemical stability of VN‐based SCs. However, the studies on oxygen‐free VN thin films, particularly those fabricated using magnetron sputtering techniques, have suggested an alternative mechanism in which the redox reactions occur directly on the nitride surface without the involvement of oxide species [92]. The exceptionally high capacitance of VN is mainly attributed to the pseudocapacitive behavior of the nitride surface rather than pure EDLC behavior [59]. This arises from the fast and reversible surface redox reactions occurring on the VN surface. Further investigation reveals a strong scan‐rate dependence of electrochemical performance. A maximum *C*_sp_ of 1340 F g^−1^ is achieved at a low scan rate of 2 mV s^−1^, which decreases to 554 F g^−1^ at 100 mV s^−1^, and still retains a capacitance of 190 F g^−1^ even at a very high scan rate of 2 V s^−1^ [22]. Notably, this capacitance exceeds that of RuO_2_ under similar conditions, highlighting the exceptional capability of VN for high performance energy storage application [59].

In addition to nanostructuring, synthesis strategies also play a vital role in influencing electrochemical performance. For instance, Glushenkov and coworkers prepared porous nanocrystalline VN via temperature‐programed ammonia reduction of V_2_O_5_, delivering a capacitance of 186 F g^−1^ at 1 A g^−1^ in KOH electrolyte [48]. To further enhance rate capability and durability, VN is frequently incorporated into composite structures and among the various categories, carbon‐based composites are most widely explored due to their large surface area, electrical conductivity, and structural stability. The incorporation of carbon‐based materials provides efficient electron transport pathways and facilitates rapid ion diffusion through porous networks with conductive materials. Ghimbeu et al. reported VN/CNT composites that retained 58% capacitance at 30 A g^−1^, compared with only 7% for pristine VN, owing to their porosity and improved electronic conductivity. Similarly, Zang et al. developed 3D VN‐functionalized CNT arrays delivering 289 F g^−1^ at 20 mV s^−1^ in 1 M KOH, and maintaining excellent rate performance even at 1000 mV s^−1^ [150]. In another study, VN nanodots embedded in carbon NSs achieved a high volumetric ED of 30.9 Wh L^−1^ and PD of 64 500 W L^−1^ [151]. Moreover, mesoporous VN NWs/CNT hybrid films showed an areal capacitance of 178 mF cm^−2^ with 82% retention after 10 000 cycles [152]. Huang et al. further demonstrated the effectiveness of carbon‐engineered VN architectures by fabricating an aqueous asymmetric SC employing CC/CW/p‐VN@C as the anode and CC/CW/Na_0.__5_MnO_2_ as the cathode. As shown in Figure 5b, CC served as a substrate for the growth of carbon wall (CW) arrays, forming a 3D CC/CW framework that enhanced surface area and electron transport. On this scaffold, carbon‐coated porous VN NSs (CC/CW/p‐VN@C) and Na_0.__5_MnO_2_ NSs (CC/CW/Na_0.__5_MnO_2_) were subsequently constructed via hydrothermal growth, nitridation/carbon coating, and electrodeposition–oxidation processes. The operating potential window of the CC/CW/p‐VN@C electrode was stably extended from −1.3 to 0 V (vs. Ag/AgCl), accompanied by a significant increase in *C*_sp_ of 604.8 F g^−1^ at 1 A g^−1^, as shown in Figure 5c,d. Furthermore, aqueous CC/CW/p‐VN@C||CC/CW/Na_0__.__5_MnO_2_ ASCs with a wide voltage window of 2.6 V were fabricated, which exhibited an ultrahigh ED of up to 96.7 W h kg^−1^ at a high PD of 1294 W kg^−1^, as shown in Figure 5e, along with outstanding cycling stability, retaining 92.5% of their capacitance after 10 000 cycles (Figure 5f) [15]. Additional systems such as VN@RGO, VN/C, VN/NCNT/NCN, and VN/NGr further confirm the beneficial role of carbon integration. As summarized in Table 2, carbon‐based VN composites consistently exhibit higher capacitance and superior rate capability compared to pristine VN due to improved conductivity and increased active surface area.

**TABLE 2:** Electrochemical performance comparison of VN‐based electrode materials for supercapacitor applications.

Materials	Capacitance	J (A g^−1^)/Scan rate (mV s^−1^)	ED	PD	Stability	Reference
MVN@NC SC	196 F g^−1^	1 A g^−1^	0.97 mWh cm^−3^	4.13 W cm^−3^	91.8% (12 000)	[153]
VN MSC	∼8 mF cm^−2^	10–100 mV s^−1^	0.3–2 µWh cm^−2^	∼10 mW cm^−2^	∼12 000 cycles	[154]
VN‐enabled SSC	231 F g^−1^	20 mV s^−1^	15.5 Wh kg^−1^	1147.3 W kg^−1^	80%, 10 000 cycles	[155]
P‐TiON//VN AMSC	72 mF cm^−2^	—	32.4 μWh cm^−2^	45 mW cm^−2^	86%, 10 000 cycles	[156]
VN/C nanoribbon SSC	266.3 F g^−1^	0.5 A g^−1^	10.3 Wh kg^−1^	—	81%, 10 000 cycles	[157]
VN nanocrystals	1340 F g^−1^	2 mV s^−1^	—	—	90%, 1000 cycles	[22]
(MVNNs)/CNT ASSSC	7.9 F cm^−3^	0.025 A cm^−3^	0.54 mWh cm^−1^	0.4 W cm^−3^	82%, 10 000 cycles	[152]
TiN@VN NWAs/CNTF FASC	328.3 mF cm^−2^	—	36.0 mWh cm^−3^	266.7 mW cm^−3^	91.5%, 5000 cycles	[158]
CC/CW/p‐VN@C ASC	604.8 F g^−1^	1 A g^−1^	96.7 Wh kg^−1^	1294 W kg^−1^	92.5%, 10 000 cycles	[15]
NiCo_2_O_4_@Ni(OH)_2_/CNTF and VN NWs/CNTF as‐fabricated FASC	291.9 mF cm^−2^	—	103.8 μWh cm^−2^	0.8 mW cm^−2^	87.2%, 5000 cycles	[159]
Co(OH)_2_//VN	62.4 F g^−1^	—	22 Wh kg^−1^	15.9 kW kg^−1^	86%, 4000 cycles	[160]
Ni/VN//Ni_1−*x*_V_*x*_O_2_ ASC	65.7 F g^−1^	2.5 mA cm^−2^	23.3 Wh kg^−1^	176.7 W kg^−1^	87%, 1000 cycles	[161]
VN	152 F g^−1^	1 A g^−1^	—	—	66%, 1000 cycles	[78]
NCQDs/VN	194.03 F g^−1^	1 mV s^−1^	—	—	—	[162]
3D carbon@TiN@VN	∼0.64 F cm^−2^	1 mA cm^−2^	—	—	—	[163]
VTiN‐4/C nanofibers	430.7 F g^−1^	0.5 A g^−1^	—	—	63%, 600 cycles	[164]
0.04‐VN/NCS_2_//NiCo_2_S_4_ ASC	65 F g^−1^	1 A g^−1^	21 Wh kg^−1^	802 W kg^−1^	74.8%, 5000 cycles	[165]
ZNCO@Ni(OH)_2_//VN@C CFASC	573.75 mF cm^−2^	—	33.66 mWh cm^−3^	—	90.3%, 3000 cycles	[166]
Ni(OH)_2_//C/VN/MnO_*x*_‐700‐1	1517 F g^−1^	1 A g^−1^	37.05 Wh kg^−1^	396.95 W kg^−1^	84%, 20,000 cycles	[167]
VN/C	386.6 F g^−1^	2 mV s^−1^	—	—	500 cycles	[168]
C/Co/VN	242.2 F g^−1^	0.5 A g^−1^	19.08 Wh kg^−1^	377.72 W kg^−1^	97%, 20 000 cycles	[169]
VN/C‐m‐5	107.77 F g^−1^	—	—	—	89.3%, 2000 cycles	[170]
VN/C‐SDS	422.0 F g^−1^	0.5 A g^−1^	—	—	45%, 80 cycles	[171]
VN/NCNT/NCN	232.9 F g^−1^	1 A g^−1^	7.3 Wh kg^−1^	549.8 W kg^−1^	91%, 5000 cycles	[172]
VN/Ni_3_N–Ni/CC	845.63 mF cm^−2^	0.5 mA cm^−2^	—	—	86.1%, 1500 cycles	[24]
MoS_2_–W_2_N//MoS_2_–VN (ASC)	200 F g^−1^	5 mV s^−1^	87.91 Wh kg^−1^	0.87 kW kg^−1^	82%, 20,000 cycles	[173]
VN–C–S	191.6 F g^−1^	1 A g^−1^	—	—	—	[55]
VN//Mo_2_N ASC	85.50 F g^−1^	—	23.3 Wh kg^−1^	2800 W kg^−1^	95.35%, 5000 cycles	[174]
VO_*x*_//VN‐ASC	44.9 F g^−1^	—	0.61 mWh cm^−3^	0.85 W cm^−3^	87.5%, 10 000 cycles	[75]
VN/CNT/Inconel/CNTs	289 F g^−1^	20 mV s^−1^	—	—	64%, 600 cycles	[150]
Ni(OH)_2_//ZIF‐8@VN ASC	278 F g^−1^	0.5 A g^−1^	49.8 Wh kg^−1^	775 W kg^−1^	82.23%, 20 000 cycles	[175]
Ni(OH)_2_//VN/C‐800‐5% ASC	214.7 F g^−1^	0.5 A g^−1^	31.36 Wh kg^−1^	774.6 W kg^−1^	92.1%, 10,000 cycles	[176]
Ni(OH)_2_//VN/NPC	90 F g^−1^	0.5 A g^−1^	32 Wh kg^−1^	398.2 W kg^−1^	91.7%, 10,000 cycles	[177]
VNNCs	407.5 F g^−1^	0.5 A g^−1^	39.29 Wh kg^−1^	775 W kg^−1^	80.59%, 10 000 cycles	[178]
VN MSC	1.2 F cm^−2^	—	25 mWh cm^−2^	4 mW cm^−2^	50 000 cycles	[147]
V_2_NT_*x*_ MXene	112.8 F g^−1^	1.85 mA cm^−2^	15.66 Wh kg^−1^	3748.4 W kg^−1^	96%, 10,000 cycles	[107]
VN@RGO	276 F g^−1^	—	4.99 Wh kg^−1^	11.06 W kg^−1^	69%, 10,000	[179]
HPCF@VNNP	240.5 F g^−1^	0.5 A g^−1^	39.3 Wh kg^−1^	4000 W kg^−1^	72.1%	[180]
VN/CNTs/Ni	—	—	102 Wh kg^−1^	105−106 W kg^−1^	—	[181]
VNQDs@PCNFs‐N/F	—	—	157.1 Wh kg^−1^	198.8 W kg^−1^	—	[182]
VN/PEDOT	226.2 F g^−1^	1 A g^−1^	48.36 Wh kg^−1^	4 kW kg^−1^	91.5%, 5000 cycles	[113]
k‐VN@C	352 mAh g^−1^	—	103 Wh kg^−1^	113 W kg^−1^	—	[183]
Ni(OH)_2_//C/Co/VN‐700−0.5	306.3 F g^−1^	0.5 A g^−1^	27.52 Wh kg^−1^	415.57 W kg^−1^	93.28%, 10,000 cycles	[184]
V_2_O_3_/VN@NFs	369 F g^−1^	5 mV s^−1^	107 Wh kg^−1^	5154 W kg^−1^	68%, 10 000 cycles	[185]
MXCF@VN	476.7 F g^−1^	1 A g^−1^	83.95 Wh kg^−1^	—	82.8%, 20 000 cycles	[186]
VN/NGr	255 F g^−1^	10 mV s^−1^	—	—	94%, 2000 cycles	[187]
VN@C NWAs ACFSS.	213.5 mF cm^−2^	—	96.07 μWh cm^−2^	270 μWh cm^−2^	96.8%, 5000 times	[108]

Collectively, these studies show that carbon integration is one of the most effective approaches for improving the electrochemical performance of VN electrodes. Carbon frameworks provide highly conductive electron transport pathways, prevent nanoparticle aggregation, increase electrolyte‐accessible surface area, and facilitate rapid ion diffusion through hierarchical porous structures. Consequently, VN/carbon composites consistently exhibit higher specific capacitance, superior rate capability, and improved cycling stability compared with pristine VN. Surface modification using CPs is another promising approach for improving the electrochemical performance of VN‐based electrodes. CPs such as PEDOT provide a conductive coating that improves electron transport while protecting VN from oxidation and dissolution, thereby enhancing cycling stability and flexibility. Chen and coworkers fabricated VN/PEDOT core–shell nanoarrays, where mesoporous VN was first formed via nitridation of VS_2_ NSs, followed by uniform PEDOT coating through electrochemical polymerization. The stepwise synthesis process is illustrated in Figure 5g. The electrode exhibits quasirectangular CV curves with weak redox peaks over a potential range of 1 V (Figure 5h), indicating dominant pseudocapacitive behavior. A high *C*_sp_ of 226.2 F g^−1^ at 1 A g^−1^ was achieved, along with high‐rate capability (175.9 F g^−1^ at 15 A g^−1^), as shown in Figure 5i. The electrode also exhibited a dominant capacitive contribution (∼93.6%), confirming fast surface‐controlled kinetics. The assembled flexible ASC operated up to 1.6 V (Figure 5j) and exhibited outstanding cycling stability, retaining 91.6% of its capacitance after 10 000 cycles, along with a high ED of 48.36 Wh kg^−1^ (Figure 5k) [113]. Overall, CP coatings primarily function as conductive and protective shells. Besides improving electrical conductivity, they suppress surface oxidation and structural degradation of VN during repeated cycling, thereby enhancing long‐term stability and mechanical flexibility without significantly sacrificing capacitance.

**FIGURE 6:**
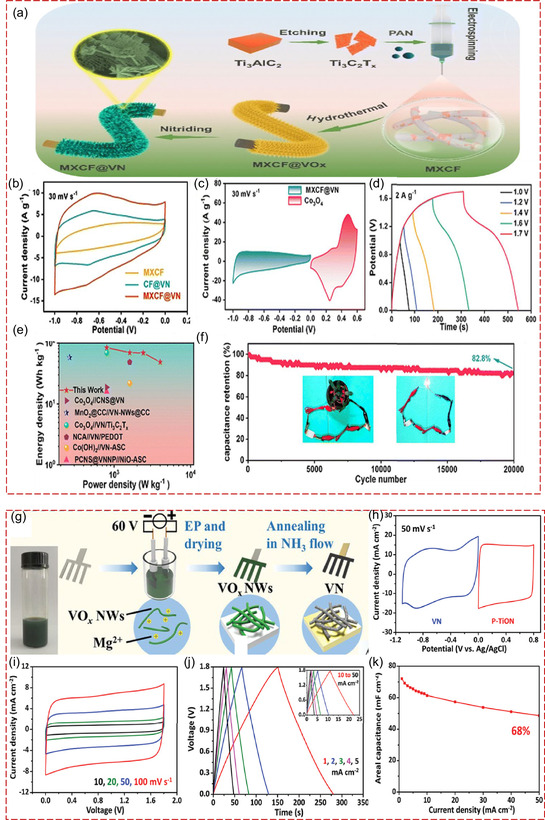
(a) Schematic illustration of the synthesis route for MXene‐derived carbon fiber‐supported VN (MXCF@VN) electrodes; (b) CV; (c) CV of MXCF@VN and Co_3_O_4_; (d) GCD; (e) Ragone plot; and (f) cycling stability and LED demonstration of the SS‐ASC. Reproduced with permission from Ref. [186]. Copyright 2024, RSC. (g) Schematic illustration of the fabrication process of the P‐TiON//VN AMSC. (h) CV curves of P‐TiON and VN nanowire microelectrodes; (i) CV; (j) GCD; and (k) areal capacitance retention of the P‐TiON//VN AMSC. Reproduced with permission from Ref. [156]. Copyright 2020, Wiley Advanced.

MXenes, a family of 2D transition metal carbide and nitrides, have gained considerable attention for SC applications due to their high electrical conductivity, hydrophilic nature, and efficient charge transport characteristics [188]. However, their electrochemical performance is often hindered by the restacking of layers, which reduces accessible surface area and restricts ion diffusion. This limitation can be effectively mitigated through hybridization with electrochemically active materials and the construction of porous or hierarchical architectures [189]. Zhang et al. prepared MXCF@VN electrodes, where MXene‐derived carbon fibers served as a conductive scaffold for the growth of VN nanoarrays, effectively preventing restacking while enhancing ion accessibility, as shown in Figure 6a. The electrode exhibited enlarged CV curves with distinct peaks over a potential window of 1 V (Figure 6b), indicating the coexistence of EDLC and pseudocapacitive behavior. It delivered a high *C*_sp_ of 476.7 F g^−1^ at 1 A g^−1^ along with good rate performance. Furthermore, the assembled solid‐state asymmetric supercapacitor (SS‐ASC) device comprising Co_3_O_4_, MXCF@VN, with a KOH/PVA gel electrolyte operated within a potential window of 1.7 V (Figure 6c) and exhibited a high ED of 83.95 Wh kg^−1^. The device also exhibited good cycling stability, maintaining 82.8% of its initial capacitance after 20 000 cycles, and could power an LED, confirming its practical applicability, as presented in Figure 6d–f, respectively [186]. These findings indicate that MXene‐based hybridization effectively overcomes the restacking issue while creating highly conductive and interconnected electrode architectures. The combined interaction between MXene and VN promotes rapid electron transport and ion diffusion, resulting in enhanced capacitance, ED, and cyclic stability. Beside the conventional SCs, MSCs have also emerged as promising miniaturized energy‐storage systems. Figure 6g further demonstrates the fabrication and electrochemical behavior of the interwoven NW‐based P‐TiON//VN AMSC. VO_*x*_ NWs were deposited and subsequently converted into conductive VN NWs through NH_3_ annealing, while PEDOT‐coated TiON NWs formed the positive electrode, creating a porous interconnected network that enhances ion diffusion and electron transport. To investigate the working voltage of the AMSC, the CV profiles of the P‐TiON NW cathode and VN NW anode were recorded at 50 mV s^−1^ in 2 M LiCl electrolyte. As shown in Figure 6h, the P‐TiON and VN NW microelectrodes exhibited stable potential windows of 0–0.8 V and −1.1 to 0 V, respectively. The CV curves maintained nearly rectangular shapes over scan rates from 10 to 100 mV s^−1^, confirming the excellent capacitive behavior of the AMSC. Figure 6i further demonstrates that the P‐TiON//VN AMSC operated stably within a voltage range of 0–1.8 V. The GCD curves shown in Figure 6j display symmetrical triangular profiles at current densities ranging from 1 to 50 mA cm^−2^, indicating good electrochemical reversibility. The corresponding areal capacitances derived from the GCD measurements are presented in Figure 6k. Notably, the AMSC delivered a high areal capacitance of 72 mF cm^−2^ at 1 mA cm^−2^, and retained 68% of its capacitance even at the current density of 50 mA cm^−2^ [156]. These reports demonstrate that the electrochemical performance of VN‐based MSCs is strongly governed by the electrode architecture. Interconnected NW networks and porous thin‐film configurations shorten ion diffusion pathways, provide continuous electron transport channels, and maximize the utilization of electrochemically active sites.

Multinitride systems such as TiN/VN improve conductivity and introduce additional redox‐active sites [158]. Metal oxide and sulfide hybrid systems are mainly used in asymmetric configurations to enhance ED, such as VN/NiOx [109] and V_2_O_3_/VN@NFs systems [185]. Thin‐film VN electrodes also provide improved stability due to reduced internal resistance [112]. Despite the excellent electrochemical performance of VN‐based electrodes, maintaining long‐term cycling stability remains a significant challenge for their practical implementation. Capacitance reduction during prolonged cycling is primarily due to the gradual surface oxidation of VN into vanadium oxides or oxynitrides, dissolution of vanadium species in aqueous electrolytes, and irreversible structural changes induced by repeated ion insertion/extraction. These processes reduce the number of electrochemically active sites and increase the internal resistance of the electrode, resulting in a gradual decline in electrochemical performance [190, 191].

The comparison in Table 2 reveals clear structure–performance relationships in the VN‐based SCs. Carbon‐based frameworks primarily enhance electrical conductivity, increase accessible surface area, and facilitate rapid ion transport, leading to improved rate capability and cycling stability. CP coatings protect the VN surface from oxidation while improving flexibility and long‐term durability. MXene‐based hybrids provide highly conductive interconnected pathways and suppress layer restacking, resulting in enhanced charge transfer kinetics and ED. Similarly, hierarchical porous nanostructures and NW architectures expose abundant electrochemically active sites and shorten ion diffusion pathways, thereby maximizing pseudocapacitive charge storage. Overall, VN‐based SCs exhibit ultrahigh *C*_sp_ up to 1350 F g^−1^ with 85%–97% pseudocapacitive contribution, high electrical conductivity of the order of 10^6^ S m^−1^, operating windows up to 2.6 V in asymmetric configurations, energy densities up to 96.7 Wh kg^−1^, and a long cycling stability of 10 000–15 000 cycles with >90% retention. These findings collectively indicate that structural engineering including nanostructuring, incorporation of conductive carbon materials, and hybridization with conductive materials significantly improves electrical conductivity and electrochemical performance of VN electrodes by improving electron transport, increasing active surface area, and mitigating structural degradation during cycling. However, this performance is highly sensitive to oxygen exposure, electrolyte selection, and operational potential window.

### 4.2 Batteries

**FIGURE 7:**
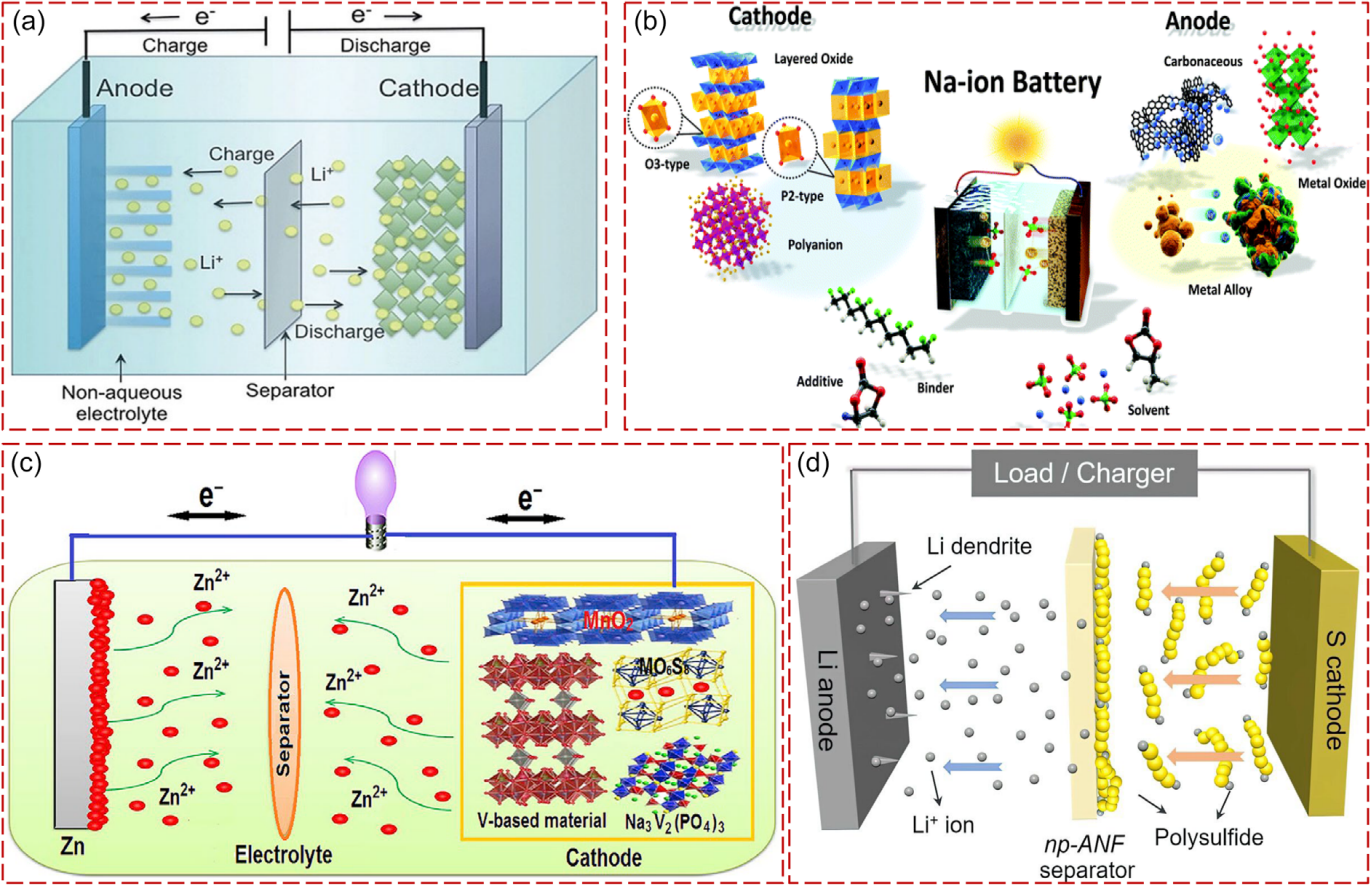
Charge storage mechanisms in (a) LIB. Reproduced with permission from Ref. [192]. Copyright 2020, MDPI. (b) NIC. Reproduced with permission from Ref. [193]. Copyright 2017, RSC. (c) ZIB. Reproduced with permission from Ref. [194]. Copyright 2020, Elsevier. (d) LSB.

Batteries store and release energy through reversible electrochemical reactions involving the transport of ions between electrodes via an electrolyte. During the charging process, metal ions are released from the cathode and transported through the electrolyte toward the anode, while during discharge, the process reverses, producing electrical energy. In LIBs, lithium ions move toward the anode during charging and intercalate into the electrode, whereas during discharging they are extracted and return to the cathode as depicted in Figure 7a. Depending on the electrode material, charge storage occurs through either intercalation or conversion reaction; conversion‐type materials undergo phase transformation to form metallic species and lithium compounds, delivering higher capacities but often causing significant volume expansion and structural deformation. In this regard, VN is considered a highly attractive electrode material for LIBs owing to its superior electrical conductivity (∼10^6^ S m^−1^), strong V—N bonding, and high theoretical capacity (∼1043 mAh g^−1^). VN stores lithium mainly through a reversible conversion reaction [195]

(VIII) $$\mathrm{VN}+3\mathrm{Li}+3e^{-}=V+{\mathrm{Li}}_{3}N$$

**FIGURE 8:**
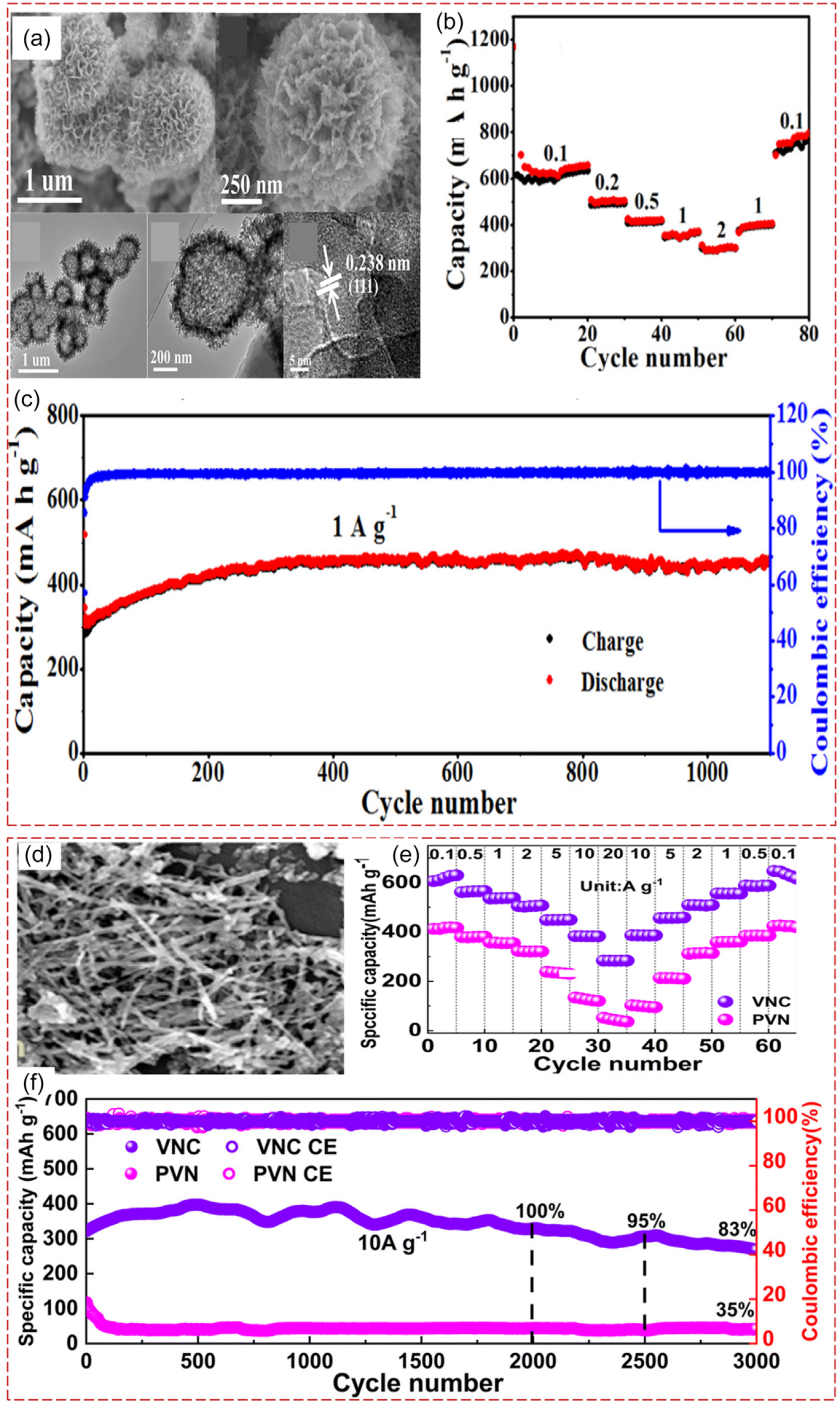
(a) Morphology; (b) CV; and (c) cyclic stability of VN hollow sphere electrodes LIBs. Reproduced with permission from Ref. [196]. Copyright 2019, Wiley Elsevier. (d) SEM of carbon‐coated VN (VNC); (e) rate performance at various current densities between 0.1 and 20 A g^−1^; and (f) cyclic performance of VNC electrodes for aqueous ZIBs. Reproduced with permission from Ref. [120]. Copyright 2024, RSC.

During lithiation, VN is reduced to metallic vanadium while lithium nitride (Li_3_N) is formed, and the reaction reverses during delithiation. The formation and disappearance of Li_3_N during cycling have been confirmed by ex situ X‐ray diffraction (XRD) analysis. However, the associated volume changes during this conversion reaction may induce structural stress and degrade long‐term cycling stability [23]. To overcome these limitations, various nanostructured VN‐based electrodes have been developed. Wang et al., developed a template‐free solvothermal method followed by ammonia annealing to synthesize VN hollow spheres, as illustrated in Figure 8a. The rate performance capacities were also reported at different current densities such as 609.3, 502.2, 418.0, 358.5, and 298.2 mAh g^−1^ at 0.1, 0.2, 0.5, 1, and 2 A g^−1^, respectively, with the capacity recovering when the current density was returned to 0.1 A g^−1^, as shown in Figure 8b, indicating strong structural stability [196]. The electrode delivered *C*_sp_ 650 mAh g^−1^ at 0.2 A g^−1^ after 200 cycles and 456 mAh g^−1^ at 1 A g^−1^ after 1100 cycles, demonstrating efficient long‐term stability as depicted in Figure 8c. Such outstanding electrochemical performance is due to the hollow interior structure that resists volume expansion and prevents structural collapse. Similarly, other VN‐based nanostructures summarized in Table 3 also demonstrate promising lithium storage performance. For instance, VN@C hollow structure delivered 495.3 mAh g^−1^ at 1 A g^−1^ with stable cycling over 500 cycles, where the carbon shell enhances conductivity and buffers volume changes [182]. Similarly, VNQDs@NrGO nanostructures exhibited 323.8 mAh g^−1^ at 2 A g^−1^ with excellent cycling stability up to 10 000 cycles, demonstrating that the conductive carbon matrices significantly improve electrochemical performance [197].

**TABLE 3:** Electrochemical performance comparison of VN and its composites‐based electrode materials for LIBs, NIBs, ZIBs, and LSBs.

Lithium‐ion batteries														
Material	Morphology/structure					Capacity, mAh g^−1^			J, A g^−1^	Cycling stability			Reference	
VN@C	Hollow VN structure supported by carbon spheres					495.3			1	500 cycles			[182]	
VNQDs@NrGO	Nanostructure					323.8			2.0	10 000 cycles			[197]	
VN	Nanolaminar					381.7			0.5	200 cycles			[198]	
Vanadium oxide–nitride	amorphous carbon matrix					220			2	200 cycles			[199]	
CC‐VN@SnS_2_	Porous					819			0.65	100 cycles			[200]	
Sodium‐ion batteries														
Material			Capacity, mAh g^−1^	J, mA g^−1^		ED, Wh kg^−1^	PD, kW kg^−1^		Cycling stability				Reference	
VN			372	50		78.43	260 W kg^−1^		7500 cycles				[28]	
VN/CNFs//NVP			257	500		—	—		—				[201]	
VN‐HC			354	50		—	—		50 cycles				[202]	
Zinc‐ion batteries														
Material			Capacity, mAh g^−1^	J, A g^−1^		ED, Wh kg^−1^			PD, kW kg^−1^	Cycling stability			Reference	
VN*_x_* O*_γ_*			200	30		129			18.5	2000 cycles at 20 A g^−1^			[203]	
VNQD/NC			498	20		—			—	—			[204]	
VN/N‐CNFs			734	0.5		534			68	30 000 cycles at 50 A g^−1^			[26]	
Rocksalt vanadium oxynitride			603	0.2 C		421			96	1500 cycles			[205]	
N_v_‐VN/C‐SS‐2			186.5	2.5		278.9			2.375	1000 cycles at 4 A g^−1^			[206]	
VN(coralVN/C)			322	0.5		—			—	95% retention, 6780 cycles at 15 A g^−1^			[207]	
VN			496	0.1		305			0.393	8000 cycles at 20 A g^−1^			[208]	
VN*_x_* O*_γ_*			231.4	1		—			—	6000 cycles at 10 A g^−1^			[209]	
NVP/VN@NC			135.4	0.1		—			—	91% retention, 2000 cycles at 5 A g^−1^			[210]	
VNTONC			261	0.5		326			261	71% retention, 1000 cycles at 2.0 A g^−1^			[211]	
VN‐V_2_O_3_			335	10		—			—	6000 cycles at 10 A g^−1^			[212]	
V_2_O_3_/VN@C			278	0.2		—			—	96% retention, 400 cycles at 200 mA g^−1^			[213]	
VVN/NC			566	0.2		386			382	85% retention, 1000 cycles at 10 A g^−1^			[214]	
VNC			630	0.1		560.41			89.19 W kg^−1^	83% retention, 3000 cycles			[120]	
Lithium–sulfur batteries														
Material		Morphology			S content, %/areal loading, mg cm^−2^			Reversible capacity, mAh g^−1^			Capacity retention	Rate property, mAh g^−1^		Reference
VN/C@TCF		Triple‐nanolayer			8.1			1417.9 at 0.1 C			0.071% decay per cycle over 300 cycles; 661.2 mAh g^−1^ after 500 cycles at 1 C	803.2 (5 C)		[215]
PCF/VN/S		Porous carbon fiber with VN nanoarrays			8.1			1310.8 at 0.1 C			1052.5 mAh g^−1^, 250 cycles	591.6 (5 C)		[216]
Co–VN/S		Microflowers			4.42			873 at 2.0 C			0.028% per cycle, 500 cycles at 2.0 C	—		[217]
VN@C		Nanowires			4.2			875			830 mAh g^−1^ after 100 cycles at 0.2 C	100 cycles at 0.2 C		[218]
Co–VN@C		Yolk−shell nanospheres			4.07			1379.2			830 mAh g^−1^ after 100 cycles at 0.2 C	300 cycles at 1 C		[219]
VN@S		—			1.0			790			145.2 mAh g^−1^ after 500 cycles at 15 C	—		[220]
Co–VN/NC		Conducting framework			4.83			1521			490 mAh g^−1^ after 1000 cycles at 2 C	1000 cycles at 2.0 C		[27]
N‐CNF@VN/HNC		Nanostructured freestanding interlayer			4			384 at 2 C			0.02% per cycle at 0.5 C	—		[221]
CoVN/C‐HS		—			—			1475 at 0.05 C			—	100 cycles at 0.2 C		[222]
VN‐NBs		Porous‐shell			5.4			5.81 mAh cm^−2^			—	632 (5.0 C)		[223]
p‐Fe_2_N/n‐VN ⊂ PNCF		Vesicle‐like nanofibers			20			16.1 at 0.1 C			0.031% per cycle for 2000 cycles at 5.0 C	500 cycles at 0.1 C		[57]
VNQD‐HG		In‐plane nanopores			—			1320 mAh g^−1^			99.95% per cycle after 500 cycles	850 mAh g^−1^ @ 4 mA cm^−2^		[224]
MB‐VN		Multibranched			6			5.47 mAh cm^−^^2^			678 mAh g^−1^ after 400 cycles at 1.0 C	707 mAh g^−1^ at 3.0 C		[203]

Sodium ion batteries operate on a similar principle to LIBs, but the larger ionic radius of Na^+^ results in slower diffusion kinetics and greater structural stress during cycling as shown in Figure 7b. VN has demonstrated promising performance in NIBs. Wei et al. reported that the charge storage mechanism of VN transitioned from a conversion‐type reaction to an intercalation‐dominated process, significantly improving long‐term cycling stability. Unlike the conventional conversion reaction that forms Na_3_N and metallic V with severe volume expansion, the tuned VN electrode exhibited a highly reversible Na^+^ insertion/extraction process with suppressed structural pulverization. The VN electrode exhibited a high capacity of 375 mAh g^−1^ at 50 A g^−1^ and maintained a stable capacity over 7500 cycles at 500 mAh g^−1^ [28]. In addition, several VN‐based composite electrodes reported in Table 3 also exhibit stable sodium storage. For instance, VN–HC composite electrodes achieved capacities of 354 mAh g^−1^ [202], while other VN nanostructures maintained stable cycling for thousands of cycles, indicating that conductive carbon matrices can enhance sodium storage kinetics and structural stability.

Aqueous ZIBs have attracted significant interest as safe and low‐cost energy storage systems because of the natural abundance of zinc, its high theoretical capacity, and the use of nonflammable aqueous electrolytes. However, sluggish reaction kinetics and structural instability of electrode materials remain major challenges. Figure 7c demonstrates the charge storage mechanism in ZIBs. VN‐based electrodes have been explored for ZIBs because of their electronic conductivity and mechanical stability. For example, VN/N‐CNF composite electrodes delivered 734 mAh g^−1^ at 0.5 A g^−1^ with excellent cycling stability over 30 000 cycles, while rock salt vanadium oxynitride electrodes demonstrated 603 mAh g^−1^ at 0.2 C, highlighting the potential of nitride‐based materials for high‐power aqueous zinc storage systems [26]. Luo et al. synthesized carbon enveloped pea‐shaped VN nanorods as an efficient cathode for ZIBs. The pea‐like nanorod architecture as shown in Figure 8d provides abundant electroactive sites and shortened Zn^2+^ diffusion pathways, while the carbon coating enhances electrical conductivity. As a result, the VNC electrode delivers a high *C*_sp_ of 630 mAh g^−1^ at 0.1 A g^−1^ as shown in Figure 8e and maintains 83% capacity retention after 3000 cycles at 10 A g^−1^, with nearly 100% coulombic efficiency (CE) as shown in Figure 8f [120].

LSBs are considered promising next‐generation energy storage systems due to their high theoretical ED. However, the dissolution and diffusion of intermediate lithium polysulfides between the electrodes, commonly referred to as the shuttle effect, result in rapid capacity decay and poor cycling stability as depicted in Figure 7d [220]. VN has been widely employed as a conductive host and polysulfide adsorbent due to its strong chemical affinity toward lithium polysulfides and high metallic conductivity. VN‐based sulfur hosts exhibit enhanced redox kinetics and effectively suppress the shuttle effect through strong V–S interactions and catalytic conversion of soluble polysulfides into insoluble Li_2_S [67]. For instance, VN/C@TCF electrodes deliver a high reversible capacity of 1417.9 mAh g^−1^ at 0.1 C and maintain 661.2 mAh g^−1^ after 500 cycles, while Co–VN/NC frameworks achieve capacities up to 1521 mAh g^−1^ with stable cycling up to 1000 cycles, demonstrating the effectiveness of VN‐based hosts in improving LSB performance [215]. Zhang et al. synthesized the VN nanobubbles (VN‐NBs) as sulfur hosts for LSBs.

**FIGURE 9:**
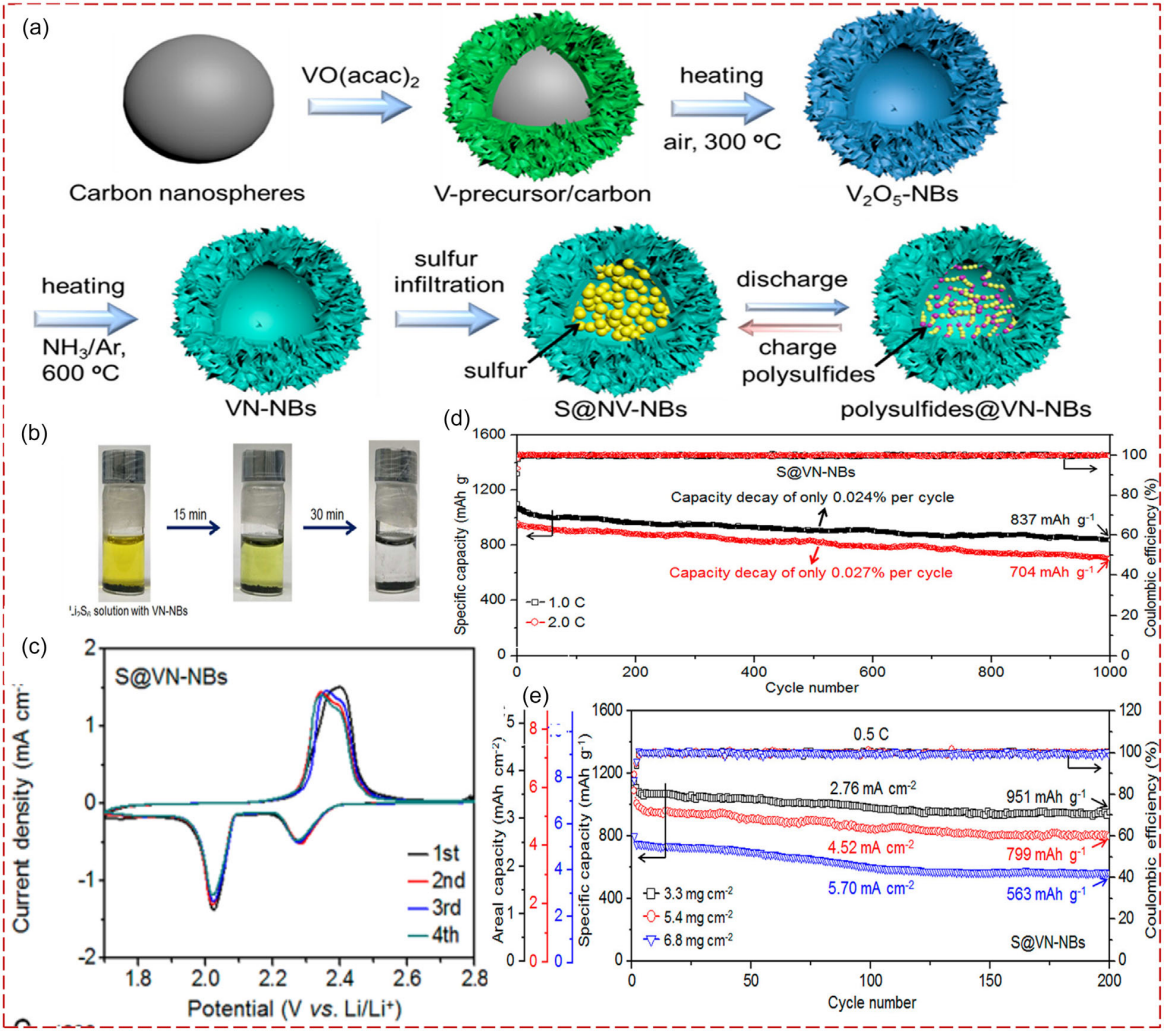
(a) VN nanobubble sulfur hosts for Li–S batteries; (b) adsorption ability test of VNNBs with Li_2_S_6_ as a representative lithium polysulfide; (c) CV curves of S@VN‐NBs cathode at 0.2 mV s^−1^; (d) long‐term cycling performances and corresponding Coulombic efficiencies of S@VN‐NBs cathodes under 1.0 and 2.0 C; and (e) long‐term cycling capabilities of S@VNNBs cathodes with sulfur loading of 3.3, 5.4, and 6.8 mg cm^−2^, respectively. Reproduced with permission from Ref. [223]. Copyright 2017, ACS.

The schematic in Figure 9a shows hollow porous VN structures that enable sulfur encapsulation and accommodate volume expansion. The adsorption test in Figure 9b confirms strong polysulfide affinity, as the Li_2_S_6_ solution becomes nearly colorless within 30 min, indicating effective suppression of the shuttle effect. Electrochemical analysis shows two distinct cathodic peaks at ∼2.28 and 2.03 V corresponding to the reduction of sulfur to long‐chain (Li_2_S_*x*_, 4 < *x *≤ 8) and short‐chain (Li_2_S_2_/Li_2_S) species, and an anodic peak at ∼2.39 V for the reverse oxidation process, indicating good redox reversibility (Figure 9c). The VN‐based cathode delivers a high initial capacity (∼1536 mAh g^−1^ at 0.2 C) and excellent rate capability (up to ∼812 mAh g^−1^ at 5 C). Long‐term cycling demonstrates remarkable stability with ∼837 mAh g^−1^ retained after 1000 cycles at 1 C and very low capacity‐decay rate (∼0.024% per cycle), along with high CE (>99%) (Figure 9d). Furthermore, even at high sulfur loadings (3.3–6.8 mg cm^−2^), the electrode maintains high areal capacities up to ∼5.81 mAh cm^−2^ and good cycling stability (Figure 9e), highlighting its practical applicability. These results confirm that VN improves conductivity, accelerates redox kinetics, and effectively confines polysulfides [223]. Table 3 summarizes the electrochemical performances of various VN and VN‐based composites used for LIBs, NIBs, ZIBs, and LSBs.

### 4.3 MICs

**FIGURE 10:**
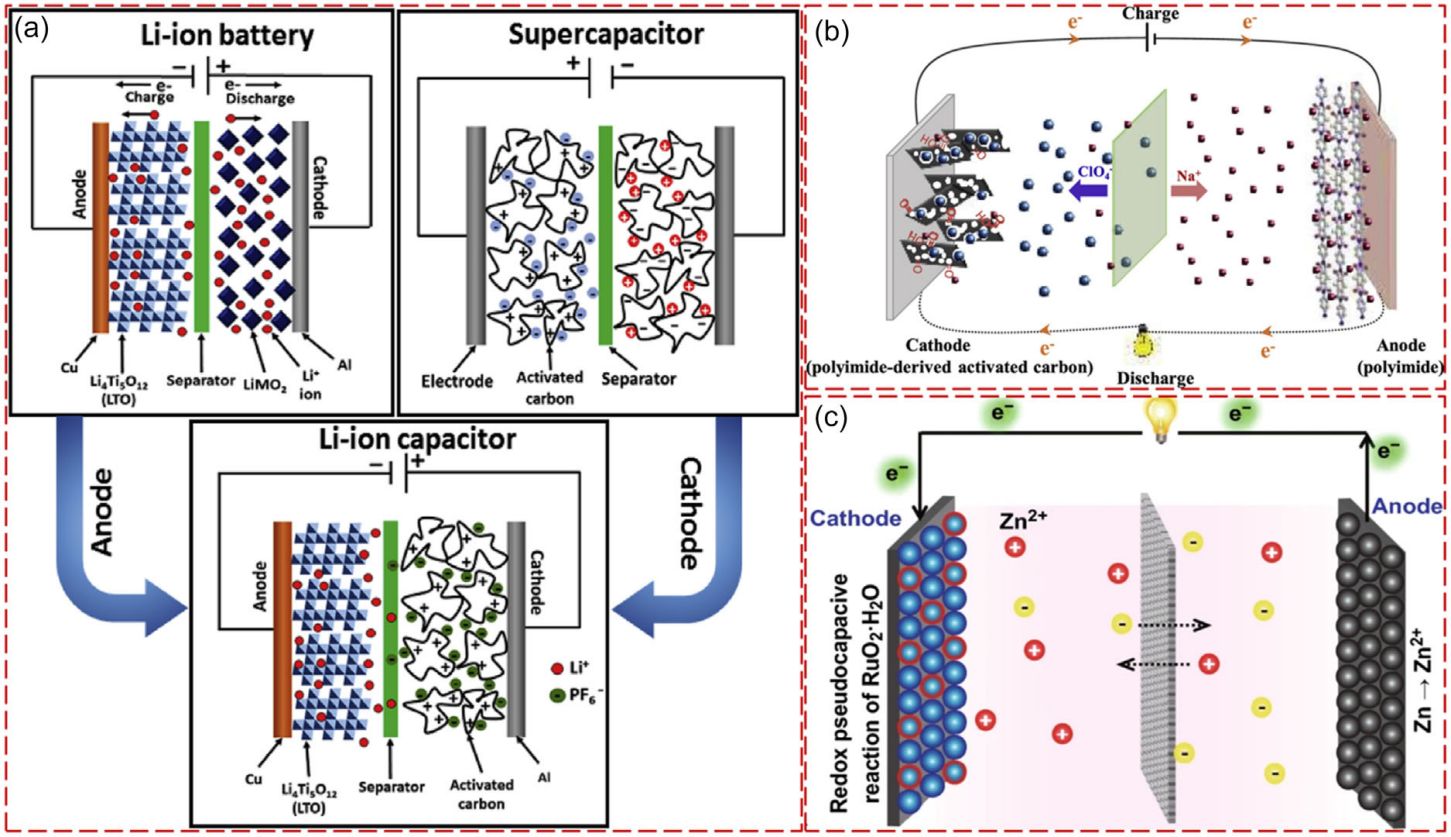
Charge storage mechanisms in (a) LICs. Reproduced with permission from Ref. [225]. Copyright 2019, Elsevier. (b) NICs. Reproduced with permission from Ref. [226]. Copyright 2018, Elsevier. (c) ZICs. Reproduced with permission from Ref. [227]. Copyright 2019, Springer Nature.

**FIGURE 11:**
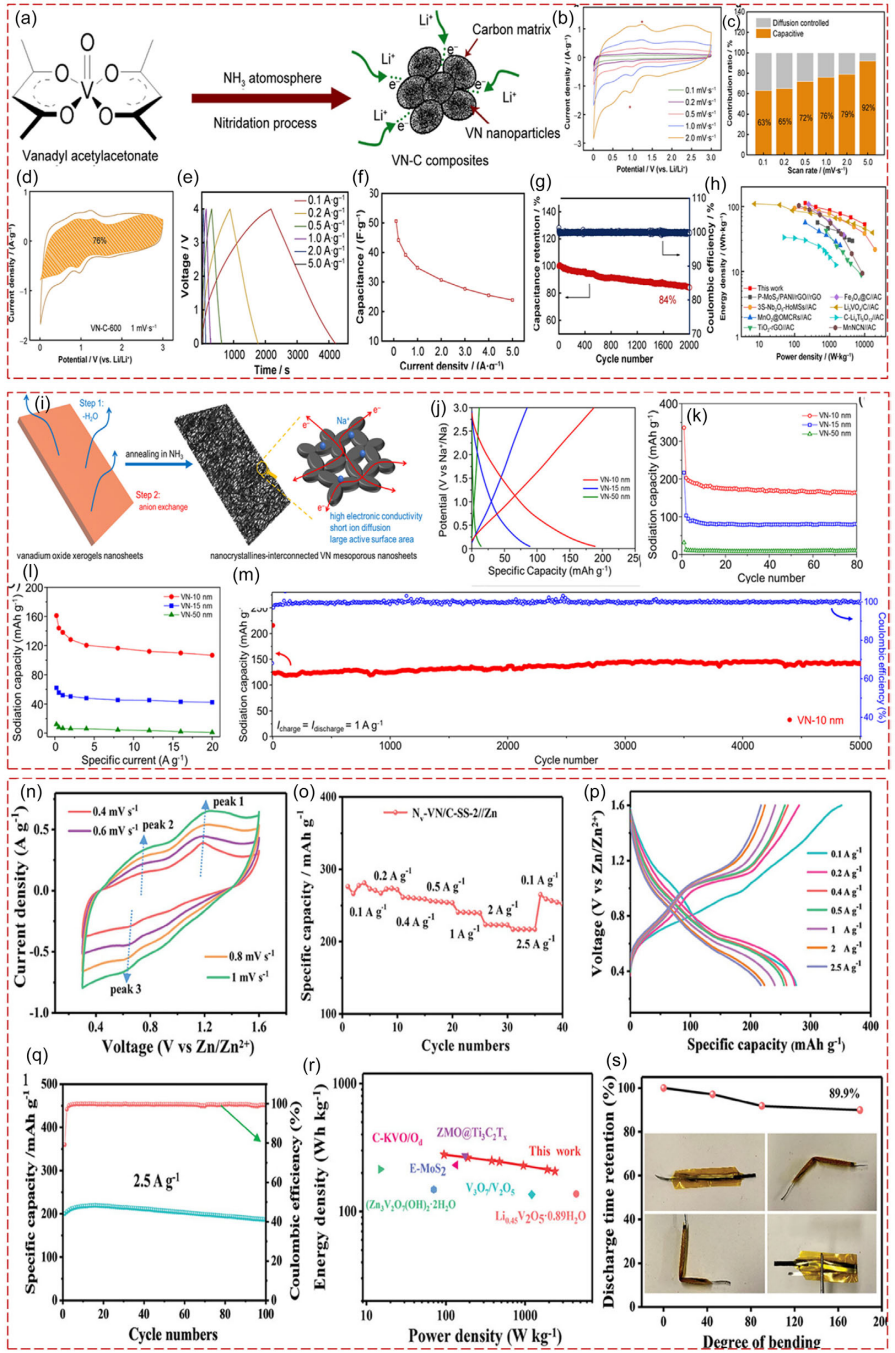
(a) Schematic illustration of VN–C; (b) CV; (c,d) capacitive and diffusion‐controlled behavior; (e) GCD; (f) capacitance at different current densities; (g) stability; and (h) Ragone plot of VN–C composites for LIC. Reproduced with permission from Ref. [239]. Copyright 2022, Springer Nature. (i) Schematic of the synthesis and nanostructure of interconnected VN mesoporous nanosheets; (j) galvanostatic charge–discharge curves at the fifth cycle; (k) cycling performance at 0.1 A g^−1^; (l) rate capability at specific currents ranging from 0.2 to 20 A g^−1^; and (m) long‐term cycling performance at 1.0 A g^−1^ for the VN‐10 nm electrode material. Reproduced with permission from Ref. [240]. Copyright 2023, Wiley Online Library. (n) CV; (o) rate performance; (p) charge–discharge curves at various current densities; (q) cycle performance; (r) Ragone plot; and (s) bending test of the Nv‐VN/C‐SS‐2//Z coin cell. Reproduced with permission from Ref. [206]. Copyright 2024, Wiley Advanced.

MICs are an advanced class of hybrid EES devices that combine the characteristics of conventional batteries and SCs. Batteries offer high ED but limited PD and cycle life, whereas SCs provide high PD and excellent cycle life but low ED (typically around 5–10 Wh kg^−1^). Therefore, a hybrid system that combines the charge storage mechanisms of both devices is required as shown in Figure 10 [228, 229]. The working principle of MICs involves a dual mechanism: a capacitor‐type cathode that stores charge through EDLC, typically using porous carbon materials such as activated carbon, carbon nanotubes, and graphene, and a battery‐type anode that stores charge via faradaic reactions using materials such as hard carbon, transition metal oxides, or alloy‐based compounds [230, 231]. During charging, the positive electrode stores charge electrostatically through ion adsorption, while the negative electrode involves a faradaic mechanism in which metal ions are inserted into the electrode structure through reversible redox reactions [232, 233]. This combined mechanism enables MICs to achieve EDs of 50–200 Wh kg^−1^, along with higher PDs than batteries and longer cycle life than pseudocapacitive SCs. The performance also critically depends on the electrode materials, electrolyte, device architecture, ion size, transport kinetics, and the electrode–electrolyte interfacial properties [233, 234]. Depending on the type of metal ion used as the anode, MICs are classified into LICs, NICs, and ZICs. Among these, LICs are the most extensively studied, and their charge storage mechanism is given in Figure 10a. Their superior performance arises from high electrical conductivity (10^3^ S m^−1^), fast Li^+^ diffusion (10^−9^ cm^2^ s^−1^), and controlled mesoporous structures (2–50 nm), which enhance ion transport and suppress dendrite formation [235, 236]. Consequently, LICs can deliver high EDs and PDs. For example, an LIC device has demonstrated an ED of 114.1 Wh kg^−1^ at 240 W kg^−1^ and retained 30.7 Wh kg^−1^ at 24 kW kg^−1^, with 78.5% capacity retention after 6000 cycles [237]. Advanced electrode architectures further enhanced LIC performance. For instance, VN/MXene‐based LIC systems exhibit high discharge capacity of 501.7 mAh g^−1^ at 0.1 A g^−1^ and 191.8 mAh g^−1^ at 5 A g^−1^, along with a maximum ED of 129.3 Wh kg^−1^ at 449.7 W kg^−1^ and outstanding cycling stability with 98% capacity retention after 5000 cycles [238]. The schematic in Figure 11a illustrates the formation of VN–C via nitridation of vanadyl acetylacetonate under an NH_3_ atmosphere, yielding VN nanoparticles embedded in a conductive carbon matrix. The CV curves (Figure 11b) exhibit quasirectangular profiles with redox peaks at ∼1.0 and 1.2 V, indicating combined capacitive and diffusion‐controlled behavior. Kinetic analysis (Figure 11c,d) shows that the capacitive contribution increases from 63% to 92% as the scan rate rises from 0.1 to 5.0 mV s^−1^, suggesting dominant surface‐controlled storage at higher rates. The GCD profiles (Figure 11e) demonstrate good rate capability, while the VN‐C‐600//p‐AC device retains ∼84% capacitance after 2000 cycles at 1 A g^−1^ with nearly 100% CE as shown in Figure 11g. The Ragone plot (Figure 11h) further reveals a high ED of 112.6 Wh kg^−1^ at a PD of 200 W kg^−1^, confirming the suitability of VN–C composites for high‐performance LICs [239]. Despite these advantages, LICs suffer from a fundamental limitation of kinetic imbalance between the rapidly responding capacitive cathode and the comparatively slow Li^+^ diffusion in the battery‐type anode, which restricts high‐rate performance. Additionally, the limited availability and high cost of lithium pose challenges for large‐scale applications [239]. To overcome these challenges, sodium‐ion capacitors (NICs) have gained significant attention as an attractive alternative energy‐storage technology. NICs combine the high ED of NIBs with the high PD of SCs, while benefiting from the abundance and low cost of sodium [241]. Similar to LICs, NICs employ a capacitive cathode and a faradaic anode as shown in Figure 10b. Their advantages include wide operating voltage, environmental friendliness, and compatibility with sustainable materials such as bio‐derived carbons. Recent studies demonstrate the effectiveness of VN in NIC systems. For example, VN–MXene NSs exhibit a high pseudocapacitive contribution (∼72.5%), enabling rapid Na^+^ storage. The electrode delivers a capacity of 372 mAh g^−1^ at 50 mA g^−1^ and maintains ∼70 mAh g^−1^ at 8 A g^−1^, with stable cycling over 10 000 cycles [1]. Wei et al. synthesized VN mesoporous NSs using thin V_2_O_5_·nH_2_O xerogels as a precursor as shown in Figure 11i. The electrochemical performance of the VN materials was evaluated using 2016‐type half‐coin cells, where metallic sodium served as both the reference and counter electrode. Among the tested samples, the VN‐10 nm anode exhibited superior sodium‐storage behavior. After 80 charge–discharge cycles at a current density of 0.1 A g^−1^, it delivered a reversible capacity of 165 mAh g^−1^, which was significantly higher than those of VN‐15 nm (81 mAh g^−1^) and VN‐50 nm (10 mAh g^−1^) as illustrated in Figure 11k. The VN‐10 nm electrode also demonstrated excellent rate capability, retaining a capacity of 106 mAh g^−1^ even at a high current density of 20 A g^−1^ (Figure 11l). Furthermore, during prolonged cycling at 1 A g^−1^ over 5000 cycles (Figure 11m), the VN‐10 nm anode maintained remarkable stability, with the *C*_sp_ gradually increasing to 144 mAh g^−1^ and achieving a CE close to 100%. These results indicate that the VN‐10 nm electrode combines high‐rate capability, long‐term cycling stability, and excellent reversible capacity, making it a highly promising candidate for high‐power sodium‐ion energy‐storage applications [240]. Similarly, VN/carbon composites show capacities of 352 mAh g^−1^ and energy densities of 103 Wh kg^−1^ with excellent cycling stability [183]. In another study, VN quantum dots embedded in porous carbon nanofibers deliver a high capacity of 392 mAh g^−1^ and ED of 157.1 Wh kg^−1^, while maintaining stability over 8000 cycles [242]. These results highlight that VN‐based anodes can effectively mitigate kinetic limitations in NICs.

ZICs represents another promising category of MIC system, consisting of a porous carbon cathode and a zinc metal anode operating in an aqueous electrolyte. They combine EDLC‐based charge storage at the cathode with reversible Zn plating/stripping at the anode as shown in Figure 10c [243]. The operation of ZICs involves charge storage primarily through EDLC at the cathode, while at the anode charge storage occurs through reversible zinc plating and stripping reactions, represented by

(IX) $${\mathrm{Zn}}^{2+}\;+\;2e^{‐}\;↔\;\mathrm{Zn}$$

During the charging process, Zn^2+^ ions gain electrons and are deposited onto the anode surface as metallic zinc, while during discharging, the deposited zinc is oxidized back into Zn^2+^ ions [244].

The total capacitance of a ZIC device is governed by the capacitances of both electrodes and can be expressed by the following relationship

(2) $$C=\frac{1}{C_{+}}+\frac{1}{C_{-}}$$

where *C*_+_ and *C*_−_ represent the capacitances of the cathode and the anode, respectively. ZICs operate at a higher voltage window, leading to improved ED compared to symmetric SCs [245].

VN‐based materials have also shown promising performance in zinc systems. Bai et al. reported nitrogen‐vacancy‐rich VN clusters embedded in a carbon matrix (Nv‐VN/C‐SS‐2) as a cathode for ZIBs. The electrode delivered a high specific capacity of 257 mAh g^−1^ at 0.2 A g^−1^ with excellent rate capability and long‐term cycling stability. When assembled into a quasisolid‐state Zn battery, Figure 11n shows the CV curves confirming a reversible two‐step Zn^2+^ intercalation/deintercalation process, while Figure 11o,p demonstrates good rate performance and stable charge–discharge behavior. Figure 11q reveals excellent cycling stability with nearly 100% CE, Figure 11r presents the high ED and PD of the device, and Figure 11s highlights its excellent flexibility, retaining 89.9% discharge‐time retention even at 180° bending [206]. However, ZICs still face challenges such as zinc dendrite formation and side reactions including HER and OER at high voltages, which limit long‐term stability [246, 247]. Table 4 gives a detailed summary of VN‐based materials employed in various MICs systems.

**TABLE 4:** Electrochemical performance comparison of VN‐based electrode materials for MICs.

Material	Type	Storage capability, mAh g^−1^	J (A g^−1^)/Scan rate (mV s^−1^)	ED, Wh kg^−1^	PD, kW kg^−1^	Stability	Reference
VN‐C‐600//p‐AC	LIC	513	0.1	112.6	200	2000 cycles	[239]
VN/N‐rGO‐5//AC	LIC	555.3	0.1	114.1	240	78.5% at 40 mV s^−1^, 6000 cycles	[237]
VN/MXene	LIC	501.7	0.1	129.3	449.7	98% at 1 A g^−1^, 5000 cycles	[238]
VN@N‐PCYS‐0.4//AC	LIC	—	—	99.7	240.0		[248]
3D VN–RGO	LIC	—	—	162	200		[230]
VN‐10nm	NIC	106	20	—	—	5000 cycles at 1 A g^−1^	[240]
VN	NIC	372	50	78.43	260	7500 cycles	[28]
VN/carbon	NIC	352		103	113	10 000 cycles	[183]
VNQDs@PCNFs‐N/F	NIC	392	0.1	157.1	95.0	73.5%, 8000 cycles	[242]
VN‐MXene	NIC	372	50 mA g^−1^				[249]
VN@CFs	KIC	245.8	0.05	49.2	10.9	86.8% after 15 000 cycles	[243]
QSS Nv‐VN/C‐SS‐2//Zn	ZIC	217.5	2.5	278.9	94.9	227 mAh g^−1^ after 1000 cycles at 4 A g^−1^	[206]
CoralVN/C	ZIC	322	0.5			95% 6780 cycles	[207]

## 5 VN for Energy Conversion Applications

Over the last few decades, sustained improvements in environmental research and sustainable energy technologies have been driven by the increasing global environmental and energy problems including environmental degradation, rapid industrialization, and the depletion of fossil fuel supplies [250, 251]. The growth of efficient energy conversion systems which convert sustainable and renewable resources into usable energy forms has drawn a lot of attention to address these issues. Due to their great efficiency, environmental friendliness and compatibility with renewable energy sources, electrochemical energy conversion technologies have become one of the most promising techniques [252, 253]. The OER, HER, ORR, CO_2_RR, and NRR are among the many electrochemical reactions that have been studied in recent years [254]. These reactions are essential for current energy conversion devices like fuel cells, metal–air batteries, and water electrolyzer. For instance, ORR controls the performance of the fuels cells and metal–air batteries, whereas HER and OER are vital half reactions in water splitting systems for sustainable hydrogen generation. While MOR is frequently used in direct methanol fuel cells for the effective conversion of chemical energy into electrical energy, CO_2_RR and NRR allow the electrochemical conversion of greenhouse gases. The interconversion of electrical and chemical energy is made easier by these electrochemical reactions that play crucial role in emerging energy conversion technologies [254]. Electrochemical energy conversion technologies provide an effective path to address global energy and environmental challenges by allowing sustainable and efficient energy utilization. Continued advances in catalyst design are essential for realizing clean and scalable energy solutions [255, 256].

### 5.1 HER

**FIGURE 12:**
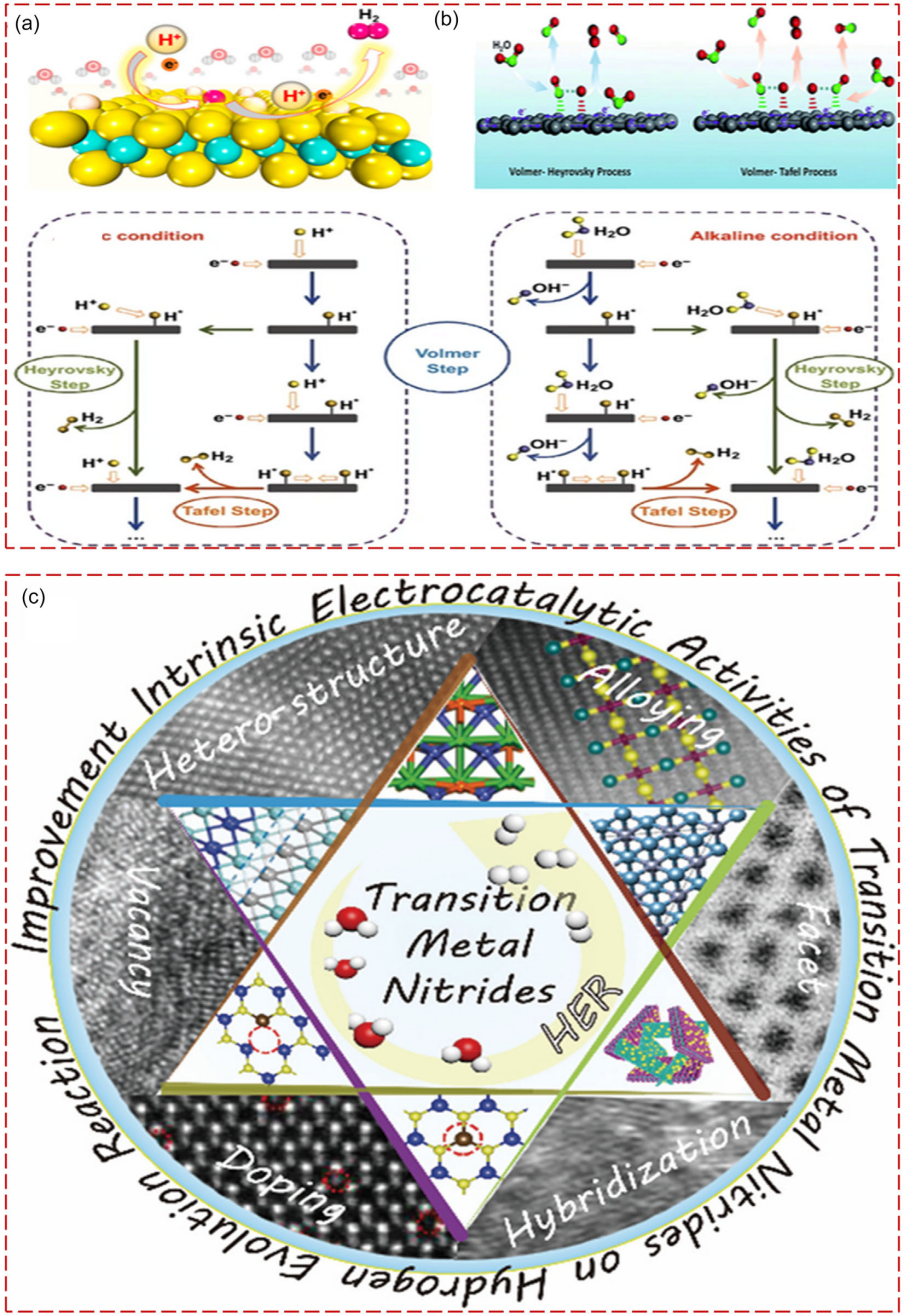
(a,b) Represent the mechanism of HER in acidic and alkaline media. Reproduced with permission from Ref. [263]. Copyright 2023, Wiley Online Library. (c) Improvement strategies for intrinsic electrocatalytic activities of TMNs on HER. Reproduced with permission from Ref. [264]. Copyright 2022, SPJ.

Molecular hydrogen has emerged as a more effective and environmentally friendly alternative to traditional fossil fuels due to significant advancements in the energy sector [257]. While there are a number of methods for producing hydrogen, including biomass electrolysis, carbonation, natural gas oxidation, steam methane reforming, and photovoltaic or electrochemical water splitting, electrochemical water splitting is widely considered as a highly promising and economically feasible method for hydrogen production [258]. Electrochemical water splitting consists of HER and OER at cathode and anode, respectively. For HER, Pt, and OER, Ru and Ir are used as state‐of‐the‐art noble electrocatalysts for efficient water splitting with low overpotential and high cost [259]. Enhancing naturally abundant electrocatalysts efficacy is another practical way to accomplish effective and affordable water splitting. The kinetics of the rate‐determining reduction step are controlled by the electronic structure of the HER catalyst. The rate of this step can be increased by using a catalyst with an ideal hydrogen binding energy [260]. The rate at which the desorbed hydrogen gas molecules are released from the catalyst surface is known as the desorption kinetics, and it is controlled by the strength of the bond between the H_2_ molecules and the catalyst surface [261]. In that, fthe majority of earth‐abundant electrocatalysts for HER are effective in both acidic and alkaline electrolytes [262]. Figure 12a,b represents the mechanism of HER in acidic and alkaline media. In acidic media, H^+^ ions are directly reduced on the catalyst surface through Volmer step to form adsorbed hydrogen (H*). This is followed by either the Heyrovsky step, where H* reacts with another proton and an electron, or the Tafel step, where two H* combine to release H_2_ gas. In alkaline media, the process is slower due to the additional water dissociation step, where H_2_O splits into H* and OH^−^. The subsequent Heyrovsky or Tafel step then produces hydrogen. Reaction efficiency depends on adsorption energy and active sites [265]. In acidic media, VN enables rapid proton adsorption through Volmer step, therefore forming H* on V sites, followed by Heyrovsky step or Tafel reactions to produce H_2_. In alkaline media, it facilitates water dissociation to generate H* and OH^−^, with N sites enhancing electronic properties and kinetics [266]. TMNs exhibit high electrical conductivity, fast charge transfer kinetics, favorable hydrogen adsorption, and excellent electrochemical stability. The intrinsic activity of TMNs can be further enhanced through heterostructure construction, alloying, facet engineering, doping, hybridization, and vacancy engineering to optimize HER performance, as shown in Figure 12c [264].

Recent years have observed a rise in interest in TMNs as advanced catalytic materials due to their remarkable characteristics and catalytic efficiency. These materials show strong structural stability, tunable electronic configurations, and high electrical conductivity that provide effective charge transfer during electrochemical reactions [34]. TMNs electronic structure is identical to that of noble metals, especially platinum, which results in equivalent catalytic performance at a substantially cheaper cost [267, 268]. These benefits have led to the emergence of TMNs as attractive materials for water splitting applications, with improved long‐term operating stability and remarkable performance in both HER and OER [18]. It is commonly known that surface characteristics, electronic conductivity, and the number of exposed sites all have a major influence on a material's catalytic activity [268]. It is essential to rationally design the structure of the VN material in order to attain good electrocatalytic performance [269]. It has been demonstrated that metal dopants can enhance the d electrons of VN nanoparticles, which is beneficial for electronic conductivity, and heteroatom N doping allows electrode materials to have both increased electrical conductivity and surface wettability [270].

**FIGURE 13:**
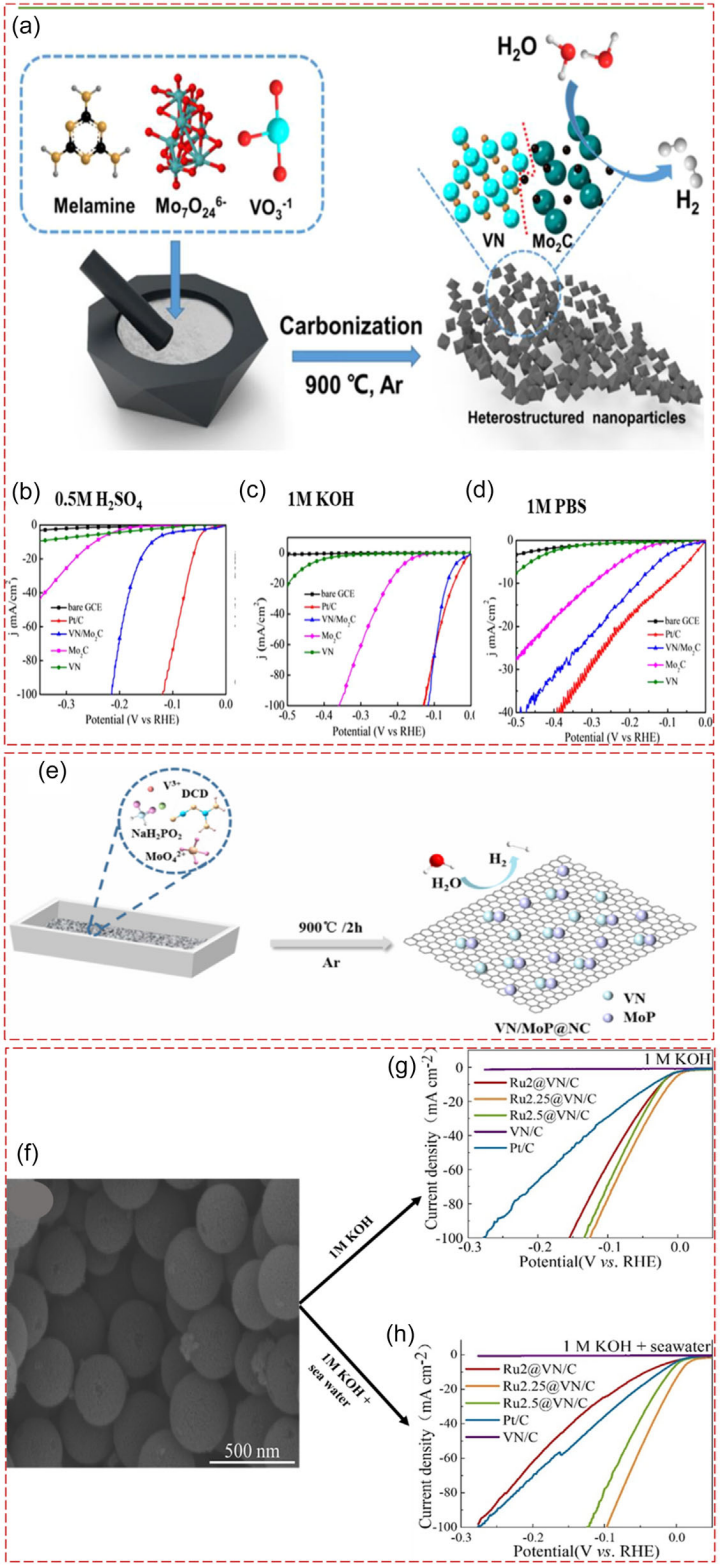
(a) Schematic illustration of synthesis of VN/Mo_2_C; (b–d) represents the LSV curve for VN/Mo_2_C at 0.5 M H_2_SO_4_, 1 M KOH and 1 M PBS solutions. Reproduced with permission from Ref. [274]. Copyright 2021, ACS. (e) Schematic representation of synthesis of VN/MoP@NC. Reproduced with permission from Ref. [275]. Copyright 2025, ACS. (f) SEM image of Ru@VN/C and (g,h) represents the LSV curve at 1 M KOH and 1 M KOH + sea water solutions. Reproduced with permission from Ref. [271]. Copyright 2024, Elsevier.

VN is a promising HER electrocatalyst due to its metallic conductivity, favorable hydrogen adsorption energy, and outstanding electrochemical stability. VN efficiently accelerates charge transfer and facilitates proton adsorption/desorption, which improves HER kinetics [271]. VN acts as an effective support and electronic modulator in heterostructure systems, resulting in synergistically improved catalytic activity and durability. Due to its lack of active sites and less advantageous hydrogen adsorption energy, pristine VN frequently exhibits low catalytic activity. The excellent HER activity of VN‐based electrocatalysts is attributed to their high electronic conductivity and favorable electronic structure, which promotes rapid electron transfer and optimize hydrogen intermediate (H*) adsorption. An ideal hydrogen adsorption free energy (ΔG_H) close to zero facilitates efficient HER through the Volmer–Heyrovsky or Volmer–Tafel mechanism. Furthermore, heterostructure formation, heteroatom doping, and defect engineering can effectively tune the electronic structure, increase active site density, and enhance catalytic activity and stability [272]. Shen et al. combined nickel and VN to develop a composite catalyst in order to get rid of these limitations. While VN offers quick electron transport, nickel enhances the water dissociation step. When compared to pristine VN, the strong interaction between Ni and VN causes a synergistic effect, improves hydrogen adsorption, increases active sites, and enhances charge transfer, which leads to significantly greater HER activity [30]. Zheng et al. used VN in this work as a lattice‐matched, highly conductive support for Ir nanoparticles, enabling robust metal–support interactions with a wide range of pH. The electronic coupling that is induced by the close lattice matching between Ir and VN optimizes the hydrogen adsorption free energy (Δ*G*_‐_H*), which speeds up the kinetics of HER. VN is an efficient electronic modulator in high‐efficiency HER systems as it reduces noble metal loading and speeds up electron transfer to Ir active sites without losing catalytic activity. The Ir–VN shows good HER activity of 21, 12, and 82 mV to reach 10 mA cm^−2^ in alkaline, acidic, and neutral conditions [273]. Feng et al. pointed out VN as a stabilizing and conductivity‐boosting element in HER catalyst during universal pH conditions. Proton adsorption and hydrogen evolution kinetics are optimized by the VN/Mo_2_C heterostructures synergistic interfacial effects in acidic, neutral, and alkaline media. Figure 13a shows schematic illustration of VN/Mo_2_C heterostructure synthesis via precursor assisted carbonization. A mixture of melamine, vanadium, and molybdenum precursor is pyrolyzed at 900 °C under Ar to form VN/Mo_2_C heterostructure nanoparticle. VN/Mo_2_C shows low overpotential of 140 mV in 0.5 M H_2_SO_4_, as shown in Figure 13b, 45 mV in 1 M KOH, as shown in Figure 13c, and 180 mV in 1 M phosphate buffered saline (PBS), as shown in Figure 13d [274]. Liu et al. showed the strong interfacial synergy between VN and MoP that enables VN/MoP@NC heterostructure to exhibit effective HER activity in alkaline media. Figure 13e shows the schematic illustration of VN/MOP@NC synthesis. Metal precursor with dicyandiamide is annealed at 900 °C under Ar to form VN/MoP nanoparticles embedded in N‐doped carbon. MoP offers beneficial hydrogen adsorption sites, while VN improves charge transfer and water dissociation. Electrical conductivity and structural stability are further enhanced by the carbon framework doped with nitrogen. The catalyst shows long‐term durability and fast reaction kinetics and low overpotential of 111 mV at 10 mA cm^−2^ in alkaline media with 50 h stability [275]. Lin et al. reported that Ru@VN/C with flower‐like mesoporous nanosphere morphology, as shown in Figure 13f, exhibits excellent HER activity in 1 M KOH and alkaline sea water as shown in the Figure 13g,h. Ru@VN/C delivered an ultralow overpotential of 5 mV and Tafel slopes of 27 mV dec^−1^ in alkaline sea water solution indicating rapid reaction kinetics and outstanding catalytic performance [271]. Table 5 shows the comparison of HER overpotential and Tafel slope of VN catalyst. The high electrical conductivity and optimal hydrogen adsorption energy of VN make it an effective HER electrocatalyst with high activity. VN is an attractive alternative to noble metal catalyst due to its performance being further improved through surface modulation, doping, and hybrid catalyst design.

**TABLE 5:** Comparison of HER overpotential and Tafel slope of VN catalyst and its hybrids.

Catalyst	Electrolyte	Overpotential, mV	Tafel slope, mV dec^−1^	Reference
Co–VN	1 M KOH	59	46.3	[276]
MoS_2_‐VN(CTAB)	0.5 H_2_SO_4_	85	53.31	[277]
Ru@VN/C	Alkaline sea water solution	5	27	[271]
MoVN films	1 M KOH	108	60	[278]
Ni_3_N‐VN	1M phosphate buffered saline	85	73	[279]
Ni_3_N@VN‐NF	1 M KOH	56	47	[280]
Ni/VN	1 M KOH	43	33	[30]
VCoN	0.1 M KOH	179		[281]

### 5.2 OER

**FIGURE 14:**
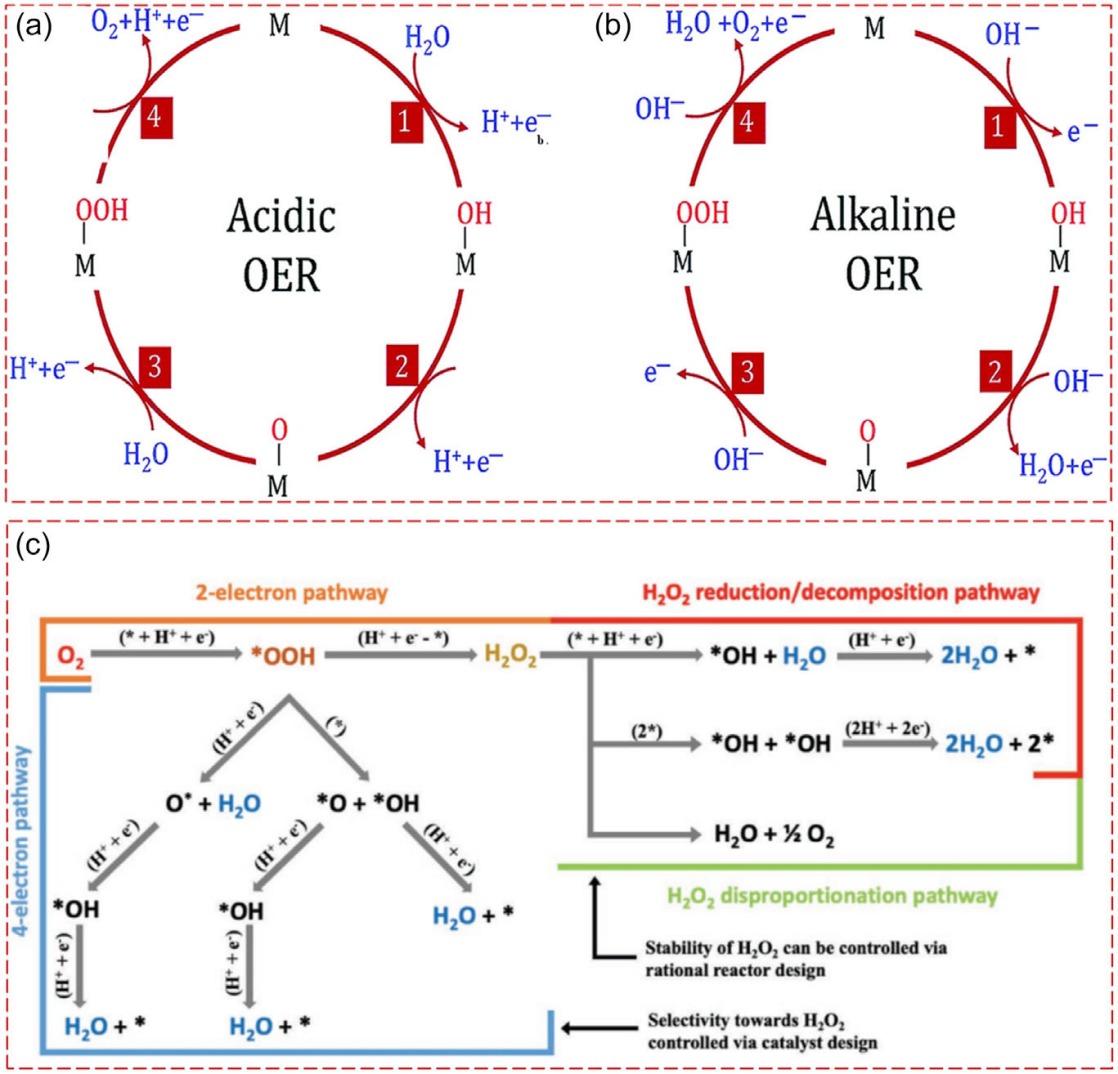
(a,b) Represent the mechanism of OER in both acidic and alkaline media. Reproduced with permission from Ref. [285]. Copyright 2022, OAE Publishers. (c) Unified two‐ and four‐electron pathway in electrochemical ORR. The two‐electron pathway (outlined in orange) proceeds via the successful preservation of the O—O bond, while dissociation of this bond would induce the undesirable four‐electron pathway forming H_2_O (outline in blue). The further reduction of H_2_O_2_ to H_2_O (outlined in red and green) is also possible following an additional four‐electron pathway. Rational catalyst are reactor design strategies that can tailor the ORR toward a selective two‐electron pathway. Reproduced with permission from Ref. [286]. Copyright 2023, Springer Nature.

The anodic OER is generally major kinetic reaction in overall water splitting applications due to its intrinsically sluggish four‐electron transfer process and complex formation of O—O bonds. It involves several reaction intermediates such as *OH, *O, and *OOH, which result in large overpotential and reduced energy efficiency, and hence it is known as the rate limiting step. The practical operation of OER requires higher potential due to kinetic limitations which results in energy loss even though thermodynamic requirement is 1.23 V in contrast to reversible hydrogen electrode (RHE) [282]. Noble metals Ru and Ir are considered as benchmark OER catalysts due to their high intrinsic activity. Due to the high cost, limited availability, and other several factors, the development of alternative OER catalysts based on earth‐abundant elements has been developed. Ni, Co, Fe, Cu, and Mn have emerged as promising candidates for OER [283]. The advantages of these materials are low cost, compositional tunability, and enhanced stability. Recently, TMNs have gained increasing attention due to their high electrical conductivity, metallic character and strong metal—nitrogen bonding which improves the rapid charge transfer and enhances catalytic stability [284]. OER proceeds through four‐step pathways in both acidic and alkaline electrolytes involving *OH, *O, and *OOH intermediates. In acidic media, H_2_O involves the release of H^+^ and electrons, while in alkaline media OH^−^ ions are involved as shown in Figure 14a,b.

**FIGURE 15:**
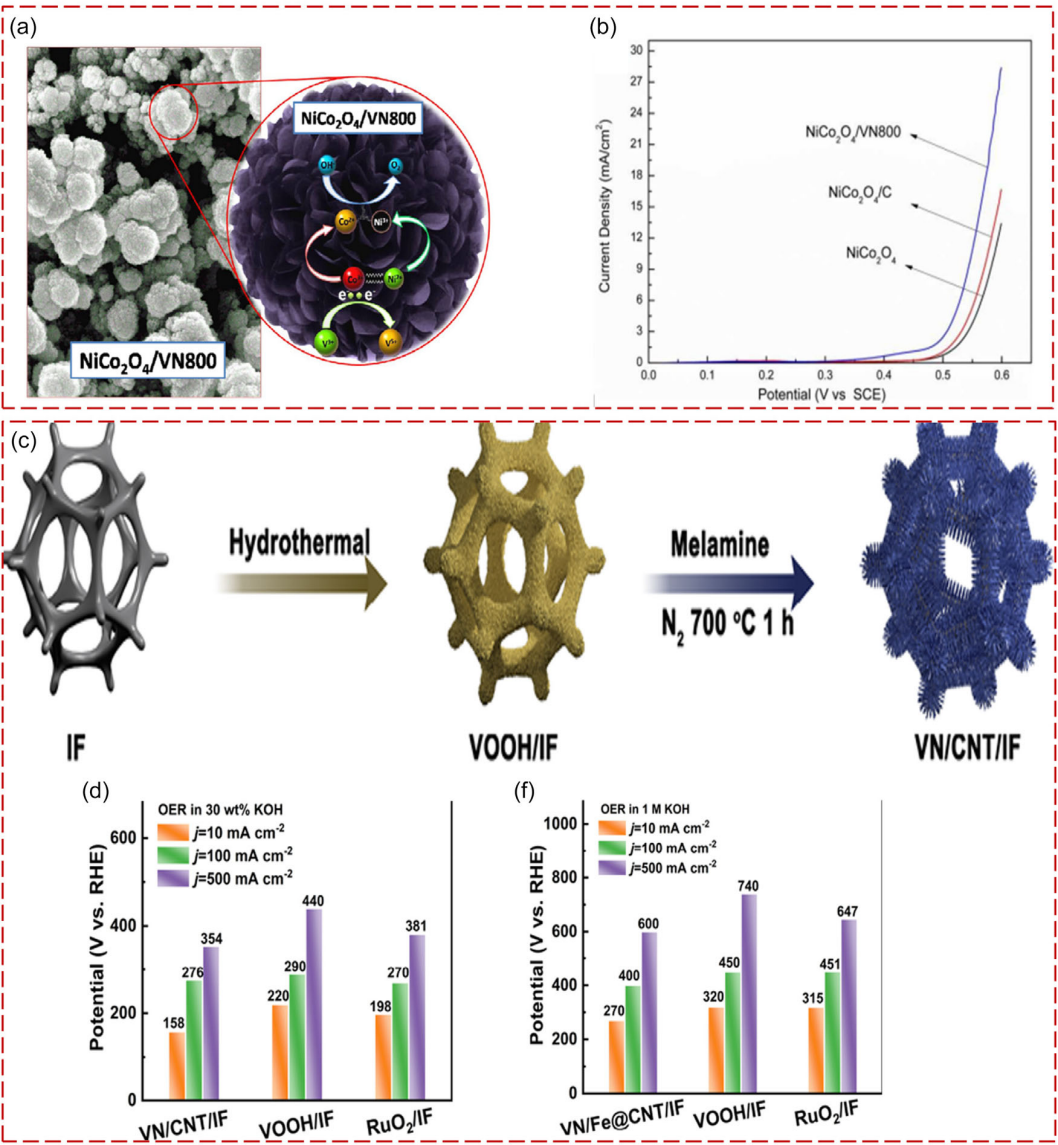
(a) Possible mechanism schematic diagram for the OER catalyzed by NiCo_2_O_4_/VN800 and (b) polarization curves for NiCo_2_O_4_, NiCo_2_O_4_/C, and NiCo_2_O_4_/VN800. Reproduced with permission from Ref. [288]. Copyright 2018, ACS. (c) Schematic illustration of the synthesis process for VN/CNT/IF and (d,e) comparison of the overpotential at different current densities in 30 wt.% KOH and 1 M KOH electrolyte. Reproduced with permission from Ref. [289]. Copyright 2022, ACS.

VN has emerged as an effective component in OER and primarily serves as a conductive support. Owing to metallic conductivity and strong metal—nitrogen bonding, VN facilitates rapid electron transport and reduced charge transfer resistance during anodic polarization. Pristine VN is a promising electrocatalyst for OER due to its increased electrical conductivity and strong chemical stability. However, the adsorption of oxygen intermediates is unfavorable and the exposed active sites are less, which limits its intrinsic catalytic activity. As a result, pristine VN is frequently altered by doping with elements like cobalt and phosphorous. These changes improve charge transfer during the reaction, inreases the number of active catalytic sites, and change the electronic structure. VN and its dopant increase catalytic efficiency, reduce overpotential, and strengthen the long‐term stability of catalyst during OER [287]. Zheng et al. showed that VN serves as a highly conductive substrate for NiCo_2_O_4_ nanoparticles, which significantly improves electron transport during OER. NiCo_2_O_4_–VN interface induces electronic coupling, which optimizes the oxidation states of Ni and Co and also enhances the formation of active Ni^3+^ and Co^3+^ oxyhydroxide species under anodic polarization. The possible mechansim for Figure 15a is attributes to interfacial electronic modulation between VN and NiCo_2_O_4_ where electrons tend to transfer from VN to the NiCo_2_O_4_ surface. Gaining the heigher oxidizing ability of Co^3+^ this electrons redistribution preferentially promotes the conversion of Co^3+^ to Co^2+^, increasing Co^2+^ content. To retain charge neutrality, the concentration of Ni^3+^ increases, thereby optimizing the electronic structure and enhancing catalytic activity.They made a composite of VN/NiCo_2_O_4_ at different temperatures of 700, 800, and 1000 °C, where NiCo_2_O_4_/VN‐800 gives better OER performance with low overpotential. The synergistic interaction between VN and NiCo_2_O_4_ lowers charge transfer resistance and accelerates OER kinetics with 385 mV of overpotential in alkaline electrolyte as shown in Figure 15b [288]. Wu et al. showed the schematic illustration of VN/CNT/IF synthesized through a hydrothermal method followed by nitridation–carbonization. Initially, vanadium oxyhydroxide precursor (VOOH) is developed on iron foam (IF) through hydrothermal method to form VOOH/IF. Later annealing with melamine under N_2_ at 700 °C converts VOOH/IF into VN/CNT/IF as shown in Figure 15c. It demonstrates that VN/CNT/IF vertical nanoarray electrode shows efficient OER performance in 30 wt% KOH electrolyte, showing an overpotential of 410 mV at 1000 mA cm^−2^ in 1.0 M KOH, giving an overpotential of 270 mV at 10 mA cm^−2^ as shown in Figure 15d,e. The catalyst shows better performance in 30 wt.% KOH electrolyte and also reported overpotential at different current densities, indicating the favorable OER kinetics facilltated by fast charge transfer processes. The vertically aligned VN/CNT provides a highly conductive and mechanically robust framework, enabling efficient electron transport and enhanced exposure of active sites [289]. Table 6 shows the comparison of OER overpotential and Tafel slope of VN catalyst. The high conductivity and structural stability of VN‐based catalysts make them highly promising for OER. In order to improve charge transfer and stabilize active oxyhydroxide species, VN primarily functions as a conductive support and electronic modulator. Hybrid‐based synergsitic VN successfully lower overpotential and enhance OER durability and kinetics.

**TABLE 6:** Comparison of OER overpotential and Tafel slope of VN catalyst and its other composites.

Catalyst	Electrolyte	Overpotential, mV	Tafel slope, mV dec^−1^	Reference
Co/VN	1 M KOH	320	50.4	[290]
CoFe–PBAs/VN	1 M KOH	290	39.72	[291]
Co/VN/NC	1 M KOH	311	113.23	[40]
VN‐NC	1 M KOH	290	95.4	[292]
Cu_2_O/CuO‐VN	1 M KOH	190	135	[31]
Co/VN	1 M KOH	320	55	[293]

### 5.3 ORR

The ORR is a crucial cathodic half reaction in electrochemical energy conversion devices including meta–air fuel cells and polymer electrolyte membrane fuel cells. These systems show slow kinetics, which include several protons‐coupled electron transfer steps, significantly restricting their performance and efficiency. The high activity of platinum‐based catalysts makes them the most efficient ORR electrocatalysts in low temperature fuel cells [294]. The low natural abundance, high expense, and low tolerance of Pt to fuel stream impurities significantly restrict its widespread commercialization. These issues have gained a lot of research toward developing a non‐Pt ORR electrocatalysts. Due to their low cost and enhanced chemical stability of metal free carbon‐based materials, they have become the most promising available alternatives.

**FIGURE 16:**
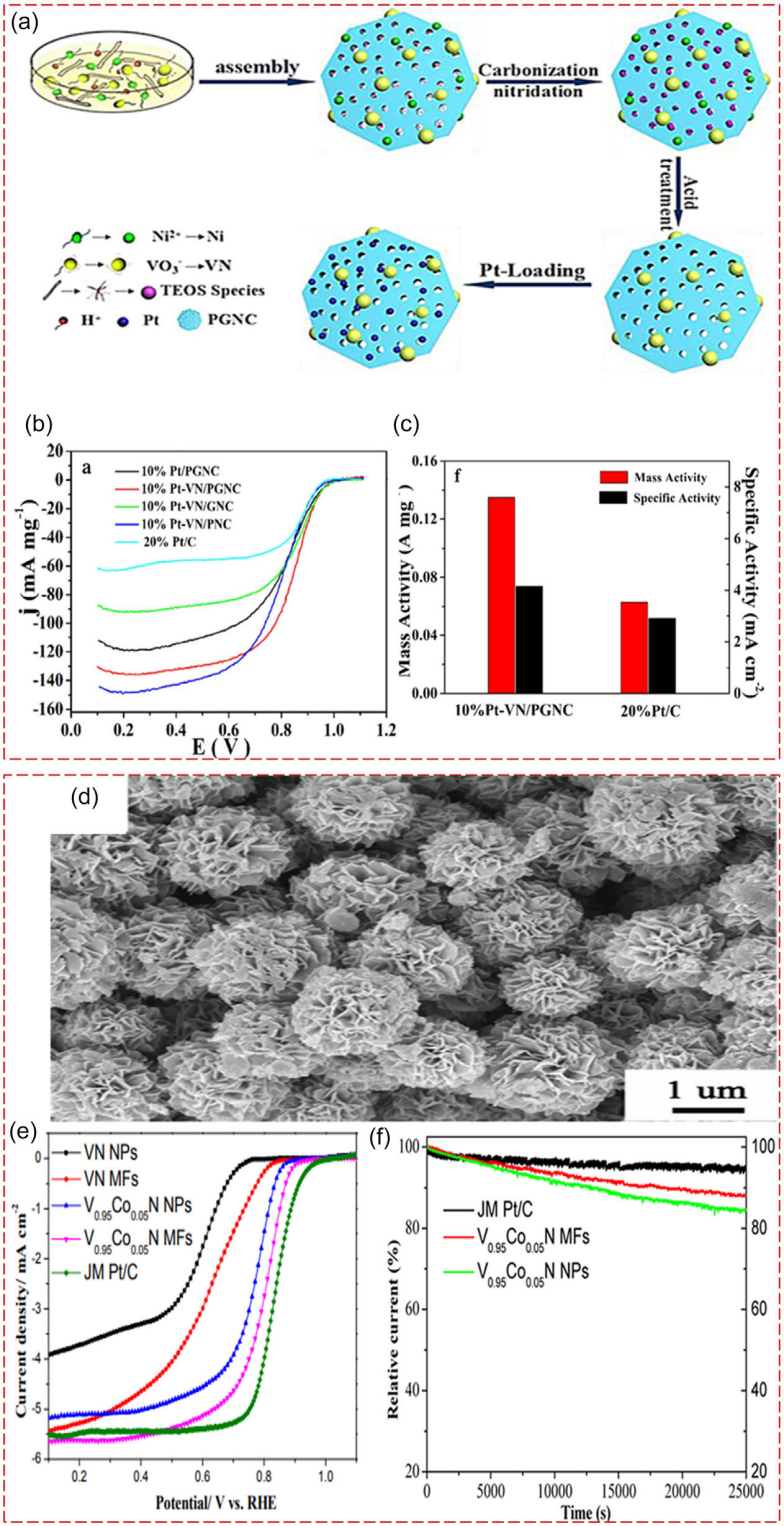
(a) Synthesis scheme followed for the construction of the Pt‐VN/PGCN composite; (b) RDE polarization curves for different materials with comparison of commercial 20% Pt/C; and (c) comparison of specific activity and mass activity. Reproduced with permission from Ref. [298]. Copyright 2015, Wiley. (d) SEM image of V_0.95_Co_0.05_O MFs; (e) linear sweep voltammetry (LSV) curves of VN NPs, VN MFs, V_0.95_Co_0.05_N NPs, V_0.95_Co_0.05_N MFs, and 20% Pt/C; and (f) catalytic stability of V_0.95_Co_0.05_N MFs, V_0.95_Co_0.05_N NPs, and 20% Pt/C polarized at 0.57 V (vs. RHE) during 25 000 s in O_2_‐saturated 0.1 M KOH solution at a rotation rate of 900 rpm. Reproduced with permission from Ref. [299]. Copyright 2018, ACS.

Techniques like heteroatom doping, defect engineering, and nanostructure design can successfully modify the electronic structure of carbon‐based materials, resulting in better ORR performance and increased oxygen adsorption [295]. ORR involves two types of mechanism, namely the four‐electron pathway and two‐electron pathway. In the two‐electron pathway, O_2_ is reduced through *OOH intermediate to produce H_2_O_2_, whereas in the four‐electron pathway O_2_ undergoes complete reduction through O and OH intermediates to form H_2_O as shown in Figure 14c [296]. VN is an efficient ORR electrocatalyst due to its metal‐like electrical conductivity and nitrogen‐induced optimized electronic structures. The modified vanadium sites speed up electron transfer and enable effective oxygen adsorption and favor the four‐electron pathway. Synergistic active sites during electrochemical operation improves catalytic stability of ORR kinetics [297]. Huang et al. showed the high electrical conductivity and noble‐metal‐like electronic structure of VN as an important ORR‐active phase in the VN/C nanocomposite system. By efficiently adsorbing and activating O_2_ molecules, the V–N framework offers numerous active sites that enhance electron transfer and support dominant four‐electron ORR pathways in alkaline media. Active site exposure is increased; charge transport is enhanced and VN agglomeration is avoided when VN nanoparticles are intimately coupled with conductive carbon. In contrast to Pt‐based catalyst, VN shows weak activity toward methanol oxidation, leading to excellent methanol tolerance. This makes VN/C ideal for alkaline direct methanol fuel cells. The result shows that VN/C exhibits considerable ORR activity achieving 75% of the diffusion limited current density and an onset potential of 0.11 V lower than that of Pt/C indicating promising catalytic performance [32]. However, surface oxidation during electrochemical reactions results in low catalytic activity for pristine VN. To overcome it, Zhang et al. investigated Co, N–Co doping to alter the electronic structure and boost active catalytic sites for ORR, that exhibited an onset potential of 1.02 V and half wave potential of 0.91 V in 1.0 M KOH solution comparable with commercial 20% Pt/C [281]. Yin et al. showed the schematic for the formation of Pt‐VN/PGNC. Initially gelatinous precursor is formed by combining Vo^3−^, Ni^2+^, glucose, and tetraethyl orthosilicate. After carbonization and nitridation followed by acid treatment VN/PGCN is formed. 10% of Pt nanoparticle is loaded on composite by borohydride reduction process to yield 10%‐Pt‐VN/PGCN composite as shown in Figure 16a. 10%‐Pt/VN/PGCN catalyst exhibits good ORR activity with 1.02 V onset potential and better catalytic stability compared with Pt/C as shown in Figure 16b. Figure 16c compares the mass and specific activities of 10%Pt‐VN/PGCN and commercial 20%Pt/C catalysts. The Pt‐VN/PGCN catalyst shows higher catalytic activity despite lower Pt loading, indicating better Pt utilization and intrinsic activity. Enhanced performance arises from the conductive VN/PGCN support which improves electron transfer and increases active sites [298]. Tang et al. showed that VN provides a strong catalytic backbone for ORR in Co‐doped VN NS‐assembled microflowers (MFs) and Co doping increases its intrinsic activity. The electronic structure of vanadium is modulated by the addition of Co to VN lattice, which optimizes the d‐band center and adjusts the adsorption strength of ORR intermediates (O_2_, OOH*, O*, and OH*). Catalytic efficiency is increased and ORR kinetics are accelerated by this electronic regulation. Further, by optimizing the exposure of VN‐based active sites and enhancing oxygen diffusion and electrolyte accessibility, the 3D porous MF structures raise ORR activity and stability as shown in Figure 16d. Figure 16e shows the comparison for VN nanoparticles (VN NPs), VN MFs, V_0.95_Co_0.05_N NPs, and V_0.95_Co_0.05_N MFs with standard Pt/C. Among these, V_0.95_Co_0.05_N MFs show the better performance nearer to standard and also showed good catalytic stability up to 25 000 s at 0.57 V (vs. RHE) in O_2_ saturated 1 M KOH solution as shown in Figure 16f [299]. Table 7 shows the comparison of ORR overpotential and Tafel slope of VN catalyst. VN is a promising non‐noble‐metal electrocatalyst for ORR due to its strong ability to activate oxygen molecules, high electrical conductivity, and noble metal like electronic structure. VN favors an effective four‐electron reduction pathway while ensuring good stability by offering optimized adsorption energies for crucial ORR intermediates. Structural engineering speeds up charge and mass transport and increases active site exposure, by coupling with conductive carbon. Transition metal doping and electronic modulation efficiently tunes the d‐band structure of VN, greatly improving ORR kinetics.

**TABLE 7:** Comparison of ORR overpotential and Tafel slope of VN catalyst and its hybrids.

Catalyst	Electrolyte	Onset potential, V	Half wave potential, V	Reference
VN/porous graphitic nanocarbon	0.1 M KOH	1.02		[298]
NGT‐CoV	0.1 M KOH	0.92		[300]
VO_*x*_N_*y*_‐CNTs	0.1 M KOH	0.10		[301]
Fe_1_NC‐V3	0.1 M KOH	1.02	0.89	[302]
V_5_NP_3_/N,P‐C	1 M NaOH	0.89	0.82	[303]
VN/NC/C‐3	0.1 M KOH	0.85	0.75	[304]
VN/C	0.1 M KOH	0.87	0.73	[32]

### 5.4 CO_2_RR

The widespread utilization of fossil fuels and their rapid rate of global industrialization have been responsible for the rise in anthropogenic CO_2_ emissions, which present serious environmental, political, and economic problems on global scale. One major cause of global warming and climate change has been found to be the swift increase in CO_2_ in the atmosphere. Out of all the different strategies, turning CO_2_ into useful chemicals and fuels is an existing and sustainable one. These approach addresses greenhouse gas concentrations while also allowing for the storage of renewable energy within chemical bonds. In this context, the electrochemical CO_2_RR has become a promising technology due to its capability to convert CO_2_ into reusable chemical fuels or feedstocks using electricity, preferably from renewable sources [305, 306]. CO_2_RR offers advantages in system design, scalability, and energy efficiency over thermochemical and photochemical methods as it can be carried out under relatively mild operating conditions within a wide range of temperature and pressure. By controlling the catalyst composition, electrolyte environment, and applied potential, it effectively increases electrocatalytic CO_2_ conversion into a variety of products. CO_2_RR has consequently emerged as key area of research for both basic and applied studies targeted as sustainable energy storage and long‐term carbon management [307]. The electrochemical CO_2_ reduction has gained increasing interest in VN as an effective electrocatalyst due to its advantageous electronic structure and metallic conductivity. Through the development of surface‐bound CO^2−^ intermediates, the partially filled d‐orbitals of vanadium allow for effective electron transfer and the activation of CO_2_ [21]. By stabilizing essential oxygen‐containing intermediates, especially *COOH, the synergistic interaction between vanadium and nitrogen sites advances the two‐electron reduction pathway that leads to the formation of CO. VN has a moderate *CO binding strength, which permits easy CO desorption while preventing additional hydrogenation. The competitive HER is effectively inhibited by the weak adsorption of hydrogen on VN surfaces, improving the selectivity of CO_2_RR. The CO_2_RR activity of VN based catalyst depends on their ability to adsorb and activate CO_2_ while stabilizing key intermediates such as *COOH, *CO, and *HCOO. Optimizing the binding energies of these intermediates through electronic structure engineering, defect creation, and heterostructure design enhances product selectivity and improves the overall CO_2_RR performance [308].

**FIGURE 17:**
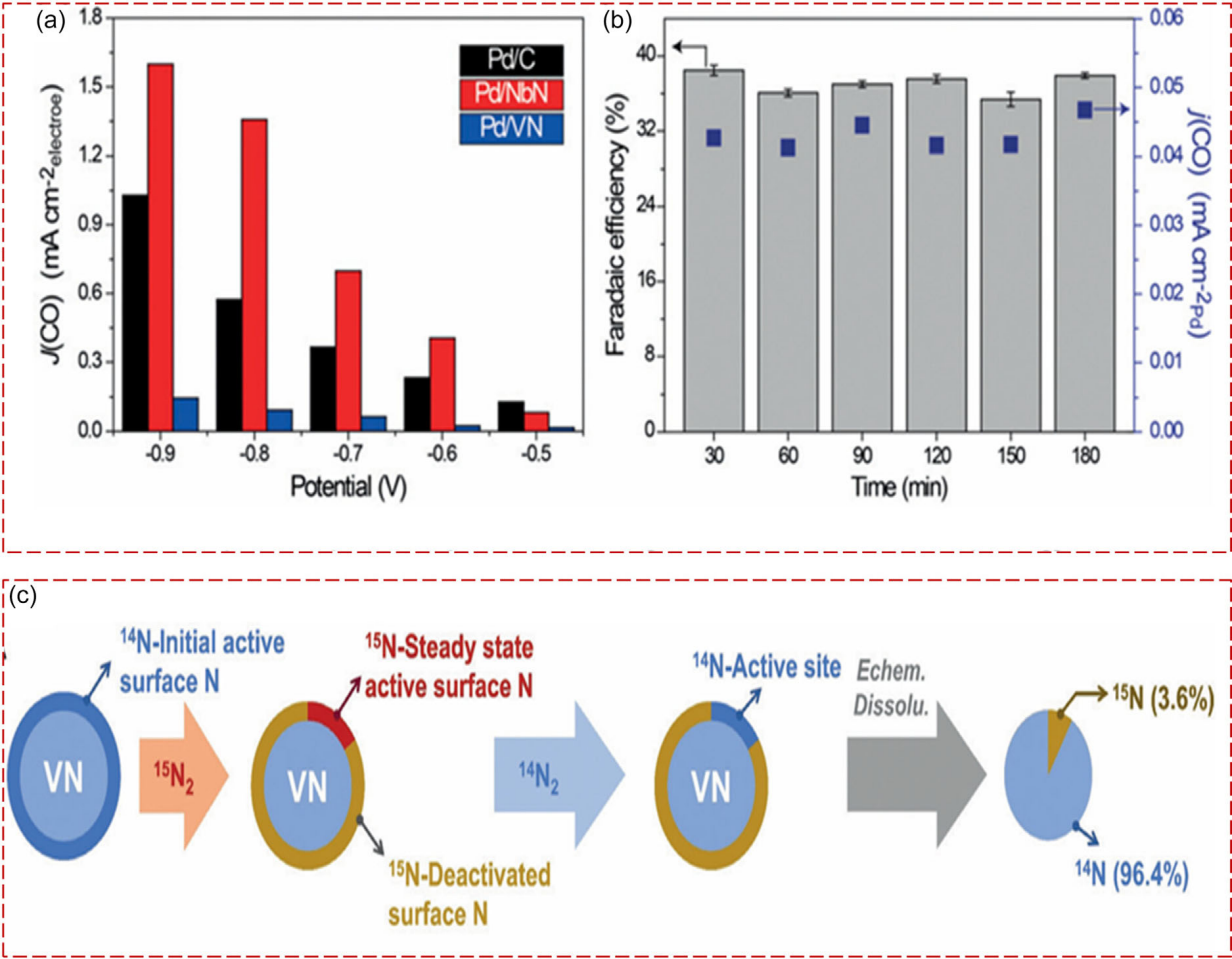
(a) CO partial current density (J(CO)) for the catalyst and (b) cycling performance of Pd/NbN at −0.6 V. Reproduced with permission from Ref. [310]. Copyright 2020, Wiley Online Library. (c) Schematic illustration of the isotropic exchange experiments on V^14^NO to determine the density of initial and steady active sites in the ENRR. Reproduced with permission from Ref. [311]. Copyright 2019, Wiley Online Library.

VN is a promising catalyst when it comes to selective and effective electrochemical CO_2_ conversion. Due to a lack of active sites, weak CO_2_ intermediate adsorption, and potential surface instability, pristine VN frequently displays low CO_2_ reduction efficiency. Improved catalytic activity and selectivity in CO_2_RR result in the formation of VN composites with conductive supports, which increase active surface area, improve electron transfer, and introduce synergistic effects. Liu et al. studies showed that in the electrochemical CO_2_ reduction reaction, TMNs are very efficient catalyst supports for regulating product selectivity. According to the study, CO_2_ adsorption, intermediate stabilization and hydrogen binding strength are all strongly influenced by strong metal–support interactions between TMNs and active metal sites [309]. This makes it possible to precisely adjust the CO/H_2_ ratio in syngas production by controlling the competition between CO_2_ reduction and the HER. According to the findings, TMNs are more than just inert supports, they are active ingredients that improve the stability, selectivity, and catalytic activity of electrochemical syngas generation [310]. Liu et al. showed the adjustable syngas ratio arising from the combined effects of catalytic activity and product selectivity highlighting the role of nitride supports in regulating CO_2_ reduction pathways as shown in Figure 17a.

Figure 17b explains that the enhanced performance originates from strong metal–support interactions where the nitride support stabilizes reaction intermediates and optimizes the active pd sites leading to improved syngas production efficiency [310]. The metallic conductivity and efficient CO_2_ activation capability of VN make it a promising CO_2_RR catalyst. The key intermediates are stabilized by the synergistic VN sites, which also inhibit the competing HER. VN helps electrochemical CO_2_ conversion by providing increased activity, selectivity and stability.

### 5.5 NRR

Overuse of fossil fuels, growing energy demands, environmental contamination, and rising greenhouse gas emissions have made energy sustainability and environmental protection the major 21st century concerns. The global economy depends on ammonia, which is also a promising carbon‐free energy carrier for hydrogen storage [312]. In order to increase crop yields, more than 80% of this chemical, which is the second most produced in the world, is used in the production of synthetic fibers, fertilizers, dyes, and nitric acid [313]. Therefore, it is crucial to develop a clean and sustainable ammonia synthesis route. Numerous methods such as chemical looping nitrogen reduction, photocatalysis, plasma‐assisted, biochemical, and nitrate electroreduction have been studied. Among these, the electrochemical nitrogen reduction reaction (ENRR) has become a viable, sustainable, and environmentally safe method of producing ammonia in ambient conditions [314]. VN‐based catalyst possess strong metal–nitrogen interactions that facilitate N_2_ adsorption and activation while lowering the energy barrier for N≡N bond cleavage. Under ambient conditions the reaction predominantly follows the associative pathway in which adsorbed intermediates such as *NNH, *NH_2_, and *NH_3_ are sequentially hydrogenated. The catalytic performance is highly dependent on the binding energies of these intermediates which determine both ammonia yield and faradaic efficiency [308, 315]. Ghosh et al.'s study shows that low‐valent vanadium centers have a strong ability to absorb N_2_ molecules, which makes them intrinsically active for nitrogen reduction. The study demonstrates how electronically adjustable vanadium sites allow for efficient π‐back donation from vanadium d‐orbitals to N_2_ antibonding orbitals, weakening the N≡N bond in the process. The results provide fundamental insights that support the use of vanadium‐based materials such as VN as promising catalysts for NRR, demonstrating that vanadium oxidation state plays a critical role in N_2_ adsorption and activation [316]. Yang et al. provide evidence that VN is an intrinsically active electrocatalyst for NRR. The measurement of surface‐active sites demonstrated a linear relationship between the rate of ammonia production and exposed VN sites. Figure 17c represents the Mars‐van Krevelen NRR mechanism on VN where lattice nitrogen in VN participates in ammonia formation and nitrogen vacancies generated during reaction are replenished by being adsorbed. According to the studies vanadium sites stabilize *N_2_H_*x*_ intermediates in a surface mediated associative pathway that drives NRR on VN. The study shows that VN is a reliable and widely accepted NRR catalyst due to its surface chemistry [311]. Due to improved N_2_ adsorption and activation at low valent vanadium sites, nitrogen vacancy‐engineered VN functions as an efficient electrocatalyst for NRR. Under ambient conditions, VN produces ammonia with good selectivity and measurable levels.

Among all the various energy storage and energy conversion applications discussed in this review, the enhanced performance of the hybrid VN‐based materials arises from the combined contributions of VN and the accompanying secondary components rather than from either constituent alone. In energy storage systems, VN serves as the primary electrochemically active phase owing to its high electrical conductivity and abundant redox‐active vanadium sites, which are responsible for the pseudocapacitive charge storage behavior. The secondary components, including conductive carbon materials, MXenes, CPs, and other transition metal compounds, primarily improve electrical conductivity, facilitate electron and ion transport, increase the accessible surface area, suppress particle agglomeration and surface oxidation, and enhance structural stability during repeated cycling. Similarly, in energy conversion applications such as HER, OER, ORR, CO_2_RR, and NRR, VN provides the intrinsically active catalytic sites through its metallic conductivity and favorable electronic structure, enabling efficient adsorption and activation of reaction intermediates. The accompanying components, including transition metal dopants, conductive carbon supports, noble metals, and heterostructure nitrides or oxides, mainly regulate the electronic structure of VN, optimize the adsorption energies of key intermediates, promote charge transfer, increase the density of exposed active sites, and improve catalytic stability. Therefore, the superior electrochemical performance of hybrid VN materials originates from the complementary and synergistic interaction between VN as the main active component and secondary materials as conductivity enhancers, electronic modifiers, or catalytic promoters. Based on the literature reviewed, the electrochemical performance of VN‐based materials can be broadly improved through five principal material design strategies: (i) nanostructure engineering to increase electrochemically active surface area and shorten ion diffusion pathways; (ii) conductive carbon integration to enhance electrical conductivity and structural stability; (iii) heterostructure and composite engineering to enhance synergistic interfacial interactions; (iv) electronic structure modulation through doping and defect engineering to optimize adsorption energies and charge transfer kinetics; and (v) interface engineering to suppress surface oxidation and improve long‐term durability. These design principles provide practical guidelines for the development of next‐generation VN‐based materials for both EES and energy conversion applications.

## 6 Conclusions and Future Perspectives

This review comprehensively summarizes recent advances on pristine VN and its composites, beginning with theoretical insights, followed by synthesis strategies, and finally discussing their diverse electrochemical applications. The synthesis of VN plays a vital role in determining its structural, morphological, and electrochemical properties. Both top‐down and bottom‐up methods have been studied; however, bottom‐up methods such as ammonolysis and hydrothermal/solvothermal synthesis offer superior control over composition, porosity, and nanostructure [35, 70]. These methods enable the design of tailored VN architectures with enhanced surface area, controlled defect density, and improved conductivity, which are essential for high‐performance electrochemical applications. For SCs applications, VN exhibits outstanding pseudocapacitive behavior due to its rapid and reversible surface redox reactions combined with metallic conductivity [140, 144]. Also, the electrochemical performance of VN is highly influenced by the electrolyte environment. Alkaline electrolytes typically provide enhanced capacitance and wider potential windows due to favorable reaction kinetics, whereas neutral or acidic electrolytes often involve ion intercalation mechanisms with comparatively limited stability. In contrast, gel and nonaqueous electrolytes have demonstrated improved cycling stability by suppressing structural degradation. These findings highlight that electrolyte engineering is as critical as material design in optimizing VN‐based SCs [59, 145]. To further enhance performance, considerable efforts have been directed towards the development of VN‐based composites. Carbon‐based materials (such as graphene, CNTs, and porous carbon) notably improve electrical conductivity, surface area, and rate capability [150, 151]. CPs (e.g., PEDOT) provide flexible coatings that enhance cycling stability and protect VN from oxidation [113]. In addition, hybrid systems involving MXenes [186], metal oxides, sulfides, and multinitride structures introduce synergistic effects by increasing active sites and enhancing charge transfer kinetics. These composite strategies effectively overcome the intrinsic limitations of pristine VN, leading to substantial progress in capacitance, ED, and durability. Beyond SCs, VN has also demonstrated strong potential in battery systems and MICs. In LIBs, VN exhibits high theoretical capacity through conversion‐type reactions, although challenges related to volume expansion and structural degradation remain [23]. Advanced nanostructuring and composite formation have been shown to mitigate these issues, improving cycling stability and rate performance. Similarly, in MICs, VN serves as an efficient electrode material that bridges the gap between batteries and SCs by delivering both high ED and PD [229, 234]. Furthermore, VN‐based materials have become promising electrocatalysts for energy conversion applications, including the HER, OER, ORR, CO_2_RR, and NRR. Their Pt‐like electronic conductivity, high conductivity, and tunable surface chemistry enable efficient adsorption and activation of reaction intermediates, making them promising low‐cost alternatives to noble‐metal catalysts. Overall, advancements in synthesis, electrolyte optimization, and composite engineering have significantly increased the electrochemical performance of VN‐based materials, highlighting their strong potential as multifunctional materials for next‐generation energy storage and conversion technologies.

### 6.1 Challenges in VN‐Based Materials

Despite its promising performance, VN still faces several critical challenges that hinder its practical and large‐scale implementation. One of the key limitations is surface oxidation and stability. VN is highly susceptible to oxidation under ambient and electrochemical conditions, leading to the formation of vanadium oxynitride layers that deteriorate electrical conductivity and reduce the number of active nitride sites. Cycling stability is another major concern, particularly in aqueous electrolytes. Repeated charge–discharge cycles induce structural distortion, surface reconstruction, and dissolution of vanadium species. These effects are more pronounced in battery‐type electrodes involving conversion reactions, where large volume changes result in mechanical stress, electrode pulverization, and rapid capacity fading. Achieving an optimal balance between high surface area and structural robustness remains challenging. Although nanostructuring increases the number of active sites, it often leads to structural instability and nanoparticle agglomeration, which negatively affect long‐term performance. In addition, VN‐based electrodes especially in aqueous systems are constrained by a limited electrochemical stability window, thereby restricting the achievable ED. Although VN electrode materials possess high theoretical capacitance and outstanding electrical conductivity, they suffer from poor electrochemical stability and rapid capacitance decline in alkaline electrolytes, presenting a major obstacle for practical applications during device evaluation. The material's performance has been primarily assessed in KOH electrolytes, indicating a limited comprehension of its behavior in other electrolyte systems and constraining the flexibility of device design. Together, these challenges highlight the necessity for innovative approaches in materials engineering and device design to fully harness the potential of VN in advanced energy storage applications. From an electrocatalytic perspective, VN still suffers from slow reaction kinetics, high overpotentials, and limited selectivity toward desired products. These limitations are primarily associated with insufficient active site exposure and suboptimal surface electronic structures. With respect to synthesis, most VN fabrication methods such as ammonolysis and high‐temperature nitridation require harsh conditions, which limit scalability, environmental sustainability, and cost‐effectiveness. Furthermore, achieving uniform morphology and high phase purity on a large scale remains difficult. Device‐level challenges also persist, including poor adhesion to current collectors, structural collapse at high mass loading, and compatibility issues with advanced electrolytes, particularly in gel or solid‐state systems.

### 6.2 Future Perspectives

**FIGURE 18:**
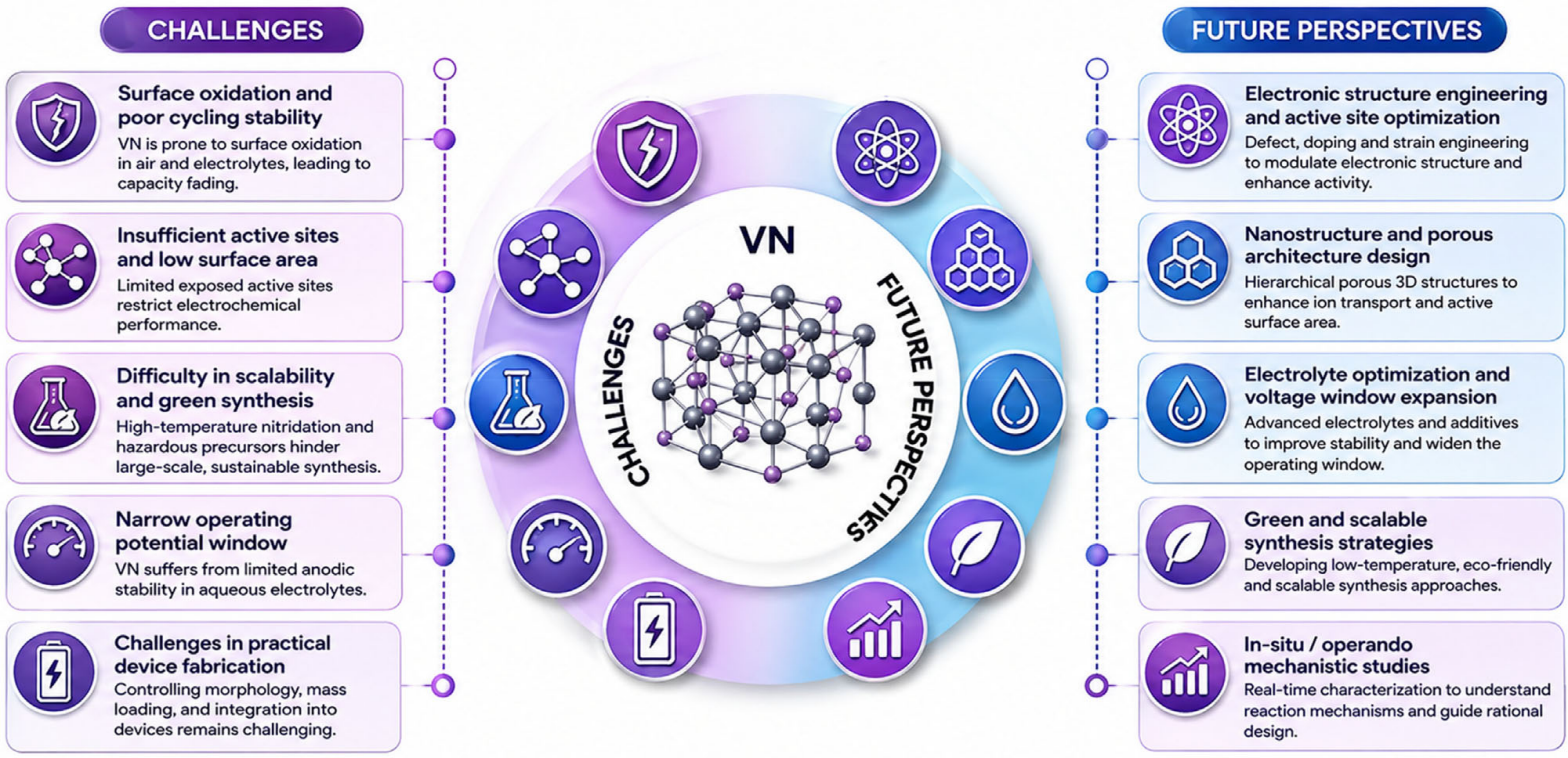
Key challenges and future perspectives for VN in energy storage and conversion applications.

To deal with those challenges, significant steps could be taken to enhance the performance of VN for both storage and conversion applications in the near future as presented in Figure 18. One of the key directions is electronic structure engineering and active site optimization. Tailoring the electronic properties of VN through doping or defect engineering can significantly enhance charge transfer and increase the number of active sites. This is particularly beneficial for SCs, where it improves capacitance and rate capability, and for catalytic processes such as HER and OER, where it reduces energy losses and enhances efficiency. Heteroatom doping, construction of heterostructures, and multimetal TMN are some of the approaches being used to modulate the electronic structure of VN‐based materials. Sun et al. demonstrated that doping of Cr^3+^ into VN induces lattice expansion, electronic structure modulation, and mesoporosity. Such doping reduces the Zn^2+^ diffusion barrier by expanding interlayer spacing and mesoporosity for ZIBs and also improves the capacitive performance by twofold relative to pristine VN [317].

Upcoming research ought to broaden this design approach to encompass a wider variety of dopants and multicomponent structures. Simultaneously, it should clarify the fundamental structure–property connections using operando characterization and multiscale modeling. Another promising strategy is the design of nanostructured and porous VN architectures. The introduction of nanoscale features and interconnected pore networks provides a large surface area and short ion diffusion pathways, which directly improve electrochemical performance in batteries and SCs while also accelerating reaction kinetics in energy conversion systems. However, these can often accelerate side reactions and structural degradation owing to their increased surface area. Electrolyte optimization and expansion of the operating voltage window are also crucial for maximizing ED and ensuring long‐term stability. Engineering advanced electrolytes represents a promising strategy for enabling VN‐based devices to operate safely at higher voltages thereby enhancing the ED of SCs. While VN exhibits significant pseudocapacitance in alkaline electrolytes, its practical use is hindered by the electrochemical instability and capacity loss of VN electrode materials. In situ electrochemical analyses indicate that the reduction in capacitance of VN from −0.4 to 0 V is due to the irreversible oxidation of vanadium (V) in N—V—O compounds by the oxygen (O) in OH^−^. The oxidized species formed are then dissolved into KOH electrolytes, compromising the electrochemical stability of VN. However, introducing a redox‐active additive (VO_4_^3−^) into the KOH electrolyte can impede this oxidation and dissolution, significantly enhancing cycling stability. These results emphasize the potential of electrolyte engineering as a strategy to address dissolution‐induced degradation in VN and highlight the importance of thoughtful interfacial design for achieving durable, high‐performance VN‐based pseudocapacitive energy‐storage devices [318]. Mass loading plays a crucial role in determining the performance of SCs. Increasing the mass loading of VN electrodes typically boosts areal capacitance by incorporating more active material. However, this often leads to reduced rate capability and cycling stability. The reasons include extended ion diffusion pathways, incomplete use of the electrode bulk, heightened mechanical stress during cycling, and faster surface oxidation or dissolution. Thus, it is crucial to optimize mass loading to strike a practical balance between energy‐storage capacity and the long‐term durability of VN‐based SCs [93]. Despite its promising performance, conventional synthesis methods typically require high temperature nitridation and controlled atmosphere resulting in high energy consumption and production costs. Maintaining consistent phase purity, morphology and nitrogen stoichiometry during scale‐up is difficult which could affect the electrochemical performance. The use of ammonia gas raises safety and environmental concerns, while surface oxidation of VN may reduce its long‐term stability. Future efforts should focus on developing low cost, energy efficient, and environmentally sustainable synthesis routes with improved scalability and reproducibility to facilitate the industrial commercialization of VN‐based materials. For instance, a bioinspired approach to creating functional metallic nitrides could be advantageous for practical wearable technologies. Yi et al. utilized naturally abundant diatomites as templates for growth, which can be applied to synthesizing various TMNs such as VN, Mo_2_N, and WN. The nitride materials’ conformal growth would replicate the morphological characteristics of the diatomite template, thus providing the resulting structures with diverse pore configurations and numerous edge defects. These metal nitrides, which exhibit excellent electrical conductivity and can be processed in solution, hold considerable potential for use in flexible printed circuits and electronic devices [155]. Furthermore, advanced in situ and operando characterization techniques will be essential for revealing the real‐time structural and electrochemical evolution of VN materials during operation providing fundamental insights for the rational design of high‐performance electrodes and catalysts. Future investigations should focus on the effective incorporation of VN‐based materials into dependable energy storage and conversion systems, such as flexible and wearable MSCs, rechargeable batteries, and high‐performance electrocatalysts for renewable energy applications. Additionally, these studies should employ integrated design methodologies that carefully balance competing factors, supported by operando analytical methods and thorough device‐level assessments under realistic mass loadings and extended cycling conditions. For example, Tang et al. successfully developed a template‐free method to create 3D porous Co‐doped VN microflowers by combining morphology control with doping effects. In an alkaline environment, this material demonstrated ORR performance on par with commercial 20% Pt/C and exhibited enhanced stability, attributed to its distinctive porous structure, thin sheets, large surface area, and charge transfer from doped Co to V atoms [299]. Overall, VN exhibits strong potential as a multifunctional material for both energy storage and energy conversion due to its tunable electronic properties, high conductivity, and adaptable nanostructures. Continuous developments in material design, scalable synthesis, electrolyte engineering, and device integration will be crucial to fully realize its practical applications in sustainable energy systems.

## Funding

This study was supported by ANRF National Post‐Doctoral Fellowship (PDF/2025/004051) and National Research Foundation of Korea (RS‐2023‐00217581, RS‐2024‐00345983).

## Conflicts of Interest

The authors declare no conflicts of interest.

## Data Availability

No primary research results, software or code have been included, and no new data were generated or analyzed as part of this review.
